# Oleanane-Type Saponins from the Roots of *Ligulariopsis shichuana* and Their *α*-Glucosidase Inhibitory Activities

**DOI:** 10.3390/molecules22111981

**Published:** 2017-11-17

**Authors:** Hai-Bo Wu, Ting-Ting Liu, Wen-Shu Wang, Jin-Chao Feng, Hong-Mei Tian

**Affiliations:** 1 College of Life and Environmental Sciences, Minzu University of China, Beijing 100081, China; whbnmr@muc.edu.cn (H.-B.W.); liutingting1204@163.com (T.-T.L.); fengjinchao@muc.edu.cn (J.-C.F.); whb860810@126.com (H.-M.T.); 2 State Key Laboratory for Biology of Plant Diseases and Insect Pests, Institute of Plant Protection, Chinese Academy of Agricultural Sciences, Beijing 100193, China

**Keywords:** *Ligulariopsis shichuana*, oleanane-type saponins, *α*-glucosidase inhibitory

## Abstract

Five new oleanane-type saponins, named ligushicosides A-E, and three known oleanane-type saponins were isolated from the roots of *Ligulariopsis shichuana*. Their structures were established by a combination of spectroscopic techniques, including 1D and 2D NMR and high resolution electrospray ionization mass spectroscopy (HR-ESI-MS). Furthermore, all isolates were evaluated for their yeast *α*-glucosidase inhibitory effects and exhibited potent inhibition against *α*-glucosidase, while compounds **1** and **2** showed excellent inhibitory activities. The 3-*O*-glycoside moiety in oleanane-type saponin is important for the *α*-glucosidase inhibitory effects.

## 1. Introduction

Nowadays, diabetes, especially type II diabetes mellitus, is a significant metabolic disorder that endangers public health, and is regarded as one of the most important public health problems in all nations [1]. One of the effective managements of type II diabetes mellitus is to retard the absorption of glucose by inhibition of carbohydrate hydrolyzing enzymes such as *α*-glucosidase in the digestive organs, which is the key enzyme catalyzing the final step in the digestive process of carbohydrates. Hence, *α*-glucosidase inhibitors can retard the liberation of glucose from dietary complex carbohydrates and delay glucose absorption, resulting in reduced postprandial plasma glucose levels and suppression of postprandial hyperglycemia. Among many classes of clinical drugs for diabetes, *α*-glucosidase inhibitors are useful for preventing the progression of the disease and for treating prediabetic conditions [2]. However, *α*-glucosidase inhibitors in clinical use usually exhibit decreased efficacy over time, ineffectiveness against some long-term diabetic complications, and low cost-effectiveness [3]. With regard to achieving and maintaining long-term glycemia control, discovering new *α*-glucosidase inhibitors is an urgent need.

Because of their perceived effectiveness, minimal side effects in clinical experience, and relatively low cost, herbal drugs are recognized as a wonderful source for medicines. Moreover, natural products have always provided new scaffolds for the design and development of new drugs, due to their structural diversity [4,5]. *Ligulariopsis shichuana* is the only species in genus *Ligulariopsis* (Compositae) that is endemic to western China. Previous studies on this plant have reported eremophilenolides and triterpenes, showing its chemical similarity with those plants in *Ligularia* [6,7,8]. Local people currently use the root and rhizome of *L. shichuana*, as well as some species of *Ligularia*, as “shanziwan”, a traditional substitute for “ziwan” (root of *Aster tataricus* L.f.) (Radix Asteris), which is a traditional Chinese medicine used to treat the symptom of xerostomia and frequent drinking caused by diabetes [9].

**Figure 1:**
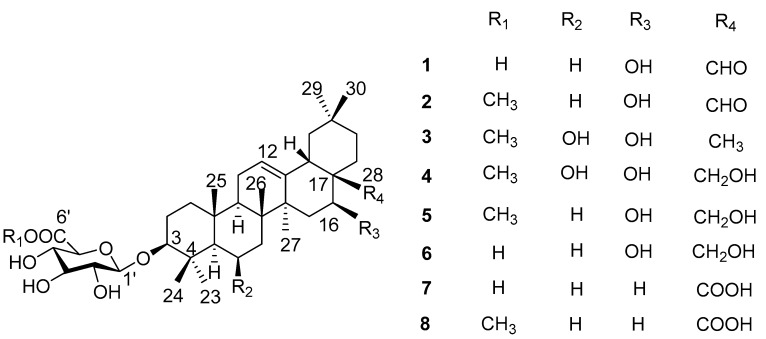
Structures of oleanane-type saponins **1**–**8** from the roots of *Ligulariopsis shichuana*.

Aiming to elucidate the antidiabetic constituents from *L. shichuana*, our team carried out phytochemical investigations on *L. shichuana*. In this study, five new oleanane-type saponins (**1**–**5**) and three known oleanane-type saponins (**6**–**8**) were found (Figure 1). All isolated compounds were tested for *α*-glucosidase inhibitory activity.

## 2. Results

Phytochemical study of MeOH extract of the roots of *L shichuana* led to the isolation of five new ligushicosides A-E (**1**–**5**) and three known oleanane-type saponins, longispinogenin 3-*O*-*β*-d-glucuronopyranoside (**6**) [10], calenduloside E (**7**) [11] and calenduloside E 6′-methyl ester (**8**) [11] (Figure 1). The known structures were identified by comparison of their spectroscopic data with those reported in the literature. Acid hydrolysis of the saponins mixture A (compounds **1**–**8**, each 1.0 mg) only gave one sugar, which was identified as glucuronic acid (GluA) by TLC, and its absolute configuration was determined as D by measuring its optical rotation [12].

**Figure 2:**
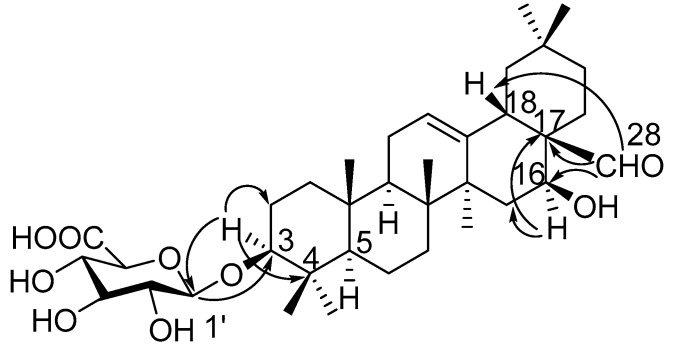
Key HMBC correlations of compound **1**.

**Figure 3:**
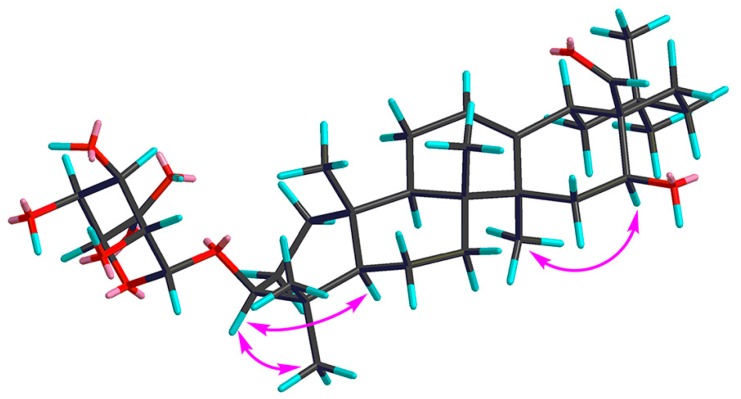
Key NOESY correlations of compound **1**.

Compound **1** was obtained as a pale yellow powder, and was found to have the molecular formula C_36_H_56_O_9_, as determined through HR-ESI-MS ([M + H]^+^ peak at *m*/*z* 633.3998, calc. for C_36_H_57_O_9_, 633.4003). IR spectrum indicated hydroxyl (3393 cm^−1^), carbonyl (1715 cm^−1^) and C=C double bond (1607 cm^−1^) functions. The ^1^H-NMR spectrum (Table 1, Figure S1) displayed signals for seven methyl groups at *δ*_H_ 0.83, 0.88, 0.96, 0.96, 0.98, 1.08 and 1.25 (each 3H, s, H_3_-26, 24, 29, 30, 25, 23, 27). Further features included signals observed at *δ*_H_ 3.19 (1H, dd, *J* = 11.7, 4.3 Hz, typical for an axial proton attached to a hydroxylate carbon), *δ*_H_ 5.34 (1H, br t, *J* = 1.5 Hz, assignable to a vinylic proton), and at *δ*_H_ 9.76 (1H, s, an aldehyde proton signal). The ^13^C NMR spectrum (Table 2, Figure S2) displayed a methine carbon signal at *δ*_C_ 208.0 (supporting the presence of a CHO group), a quaternary carbon signal at *δ*_C_ 175.5 (ester carbon), and two olefinic carbon signals (one quaternary at *δ*_C_ 142.0 and one methine at *δ*_C_ 123.1, suggesting the presence of a double bond). Furthermore, the spectrum also showed a signal at *δ*_C_ 105.3 (CH) assignable to an anomeric carbon in a sugar unit. On the basis of these data, compound **1** was determined to be a triterpene glycoside. Its aglycone was determined to be gummosogenin [13], due to their similar NMR data, except for the presence of one set of resonances attributable to a *β*-d-glucuronopyranosyl moiety [H-1′ (*δ*_H_ 4.38, 1H, d, *J* = 8.2 Hz), *δ*_C_ 74.1, 75.3, 76.6, 78.1, 105.3 and 175.5] in **1** [14]. Comparison of the ^13^C NMR data of **1** with that of gummosogenin showed the downfield shift of C-3 (+11.4 ppm) and upfield shift of C-2 (−2.6 ppm) in **1**, indicating glycosylation at C-3. This assignment was further confirmed by the HMBC correlations (Figure 2, Figure S5) between H-3 (*δ*_H_ 3.19, 1H, dd, *J* = 11.7, 4.3 Hz) in the aglycon and C-1′ (*δ*_C_ 105.3) in *β*-d-glucuronopyranosyl moiety, and between H-1′ and C-3. The relative configuration of **1** was confirmed by nuclear overhauser enhancement spectroscopy (NOESY) (Figure 3, Figure S6). The *α*-orientation of H-3 was deduced from the correlations of H-3 with H-5 (*δ*_H_ 0.81, 1H, m) and H_3_-23 respectively. The *α*-orientation of H-16 (*δ*_H_ 4.32, 1H, dd, *J* = 11.9, 4.5 Hz) was deduced from the correlations of H-16 with H_3_-27. The structure of **1** was confirmed by a complete acid hydrolysis of the saponin mixture A, which afforded D-glucuronic acid, as identified by co-TLC with an authentic sample [12]. On the basis of these findings, the structure of **1** was elucidated and named ligushicoside A.

The structure determination of compound **2** was performed in a similar manner to that described for **1**. Its molecular formula was determined as C_37_H_58_O_9_ on the basis of HR-ESI-MS ([M + Na]^+^ peak at *m*/*z* 669.3972, calc. for C_37_H_58_O_9_Na, 669.3979) and NMR experiments. Compared with the NMR data of **1**, the ^1^H NMR spectrum of **2** (Table 1, Figure S7) displayed a signal for an additional methoxy group at 6′-OCH_3_ (*δ*_H_ 3.77, 3H, s), and the sugar carbon data revealed a noticeable difference in the *δ* value of C-6′ (−5.5 ppm). This evidence, together with the observed correlation between 6′-OCH_3_ and C-6′ (*δ*_C_ 170.0) in its HMBC (Figure S12), suggested that there was a 6′-*O*-methyl-*β*-glucuronopyranoside in compound **2 [15]**. The relative configuration of **2** was confirmed by the NOESY spectrum, which was identical to that of **1**, thus permitting assignment of the molecular structure of **2**, and its naming as ligushicoside B.

The HR-ESI-MS ([M + Na]^+^ peak at *m*/*z* 671.4128, calc. for C_37_H_60_O_9_Na, 671.4135) of compound **3** established a molecular formula of C_37_H_60_O_9_. The ^1^H (Table 1, Figure S14) and the ^13^C-NMR (Table 2, Figure S15) spectra of **3** were very similar to those of **2**, except for the disappearance of the aldehyde group and the appearance of a methyl group and a hydroxy group. The HMBC correlations (Figure S18) between H_3_-28 (*δ*_H_ 0.79, 3H, s) and C-16 (*δ*_C_ 66.4), C-17 (*δ*_C_ 38.6), C-18 (*δ*_C_ 50.8), and C-22 (*δ*_C_ 31.7) indicated that the C-28 aldehyde group in **2** had been changed into a methyl group in **3**. The second hydroxy group in **3** was assigned to C-6 (*δ*_C_ 68.6), due to the COSY correlation (Figure S16) between the protons at H-6 (*δ*_H_ 4.51, 1H, br s) and H-5 (*δ*_H_ 0.75, 1H, m), and between those at H-6 and H_2_-7 (*δ*_H_ 1.55, 1H, m; 1.72, 1H, m), as well as the HMBC correlation. The relative configuration of **3** was confirmed by its NOESY spectrum (Figure S19), which was identical to that of **1**. Furthermore, the *β*-orientation of OH-6 was deduced from the correlations of H-6 and H-5, H_3_-23 (*δ*_H_ 1.13, 3H, s), and the *α*-orientation of H-16 (*δ*_H_ 4.13, 1H, dd, *J* = 11.4, 4.5 Hz) was deduced from the correlations of H-16 and H_3_-27 (*δ*_H_ 1.19, 3H, s) in the NOSEY spectrum. Thus, the structure of ligushicoside C was assigned to **3**.

The molecular formula of compound **4** was established as C_37_H_60_O_10_ ([M + Na]^+^ peak at *m*/*z* 687.4076, calc. for C_37_H_60_O_10_Na, 687.4084) by the HR-ESI-MS and NMR data. The ^1^H (Table 1, Figure S21) and ^13^C NMR (Table 2, Figure S22) spectra of **4** were similar to those of olean-12-en-3*β*,6*β*,16*β*,28-tetraol, previously obtained from this plant [8]. The presence of a 6′-*O*-methyl-*β*-glucuronopyranoside moiety [H-1′ (*δ*_H_ 4.37, 1H, d, *J* = 7.8 Hz) and 6′-OCH_3_ (*δ*_H_ 3.76, 3H, s); *δ*_C_ 52.8, 73.3, 75.4, 76.7, 77.6, 107.1 and 171.5] in **4**, which was attached to the C-3 (*δ*_C_ 91.3) supported by the glycosylation shift at the C-3 position (+11.5 ppm) in its ^13^C NMR spectra. On the basis of these findings, the structure of **4** was elucidated and named ligushicoside D.

The molecular formula of compound **5** was determined as C_37_H_60_O_9_ ([M + Na]^+^ peak at *m*/*z* 671.4128, calc. for C_37_H_60_O_9_Na, 671.4135) by its HR-ESI-MS and NMR data. The NMR signals (Table 1 and Table 2, Figures S28 and S29) of **5** were similar to those of longispinogenin 3-*O*-*β*-d-glucuronopyranoside [10], except for an additional methoxy group at 6′-OCH_3_ (*δ*_H_ 3.79, 3H, s). The HMBC correlation (Figure S32) between 6′-OCH_3_ and C-6′ (*δ*_C_ 171.5) suggested that there was a 6′-*O*-methyl-*β*-glucuronopyranoside in compound **5**. Thus, **5** was named ligushicoside E.

All isolated compounds **1**–**8** were tested for *α*-glucosidase inhibitory activity. The IC_50_ values, defined as the compound concentration that inhibits *α*-glucosidase activity by 50%, are summarized in Table 3. The results showed that all of the oleanane-type saponins isolated from the roots of *L. shichuana* displayed inhibitory activity against *α*-glucosidase, with IC_50_ values in the range of 18.7–154.3 μM, which was obviously stronger than the positive control of acarbose (IC_50_ = 190.5 μM). This result was in agreement with a report suggesting that the 3-*O*-glycoside moiety was essential to the inhibition activity of oleanane-type glycosides [16]. Furthermore, compounds **1** (IC_50_ = 18.7 μM), **6** (IC_50_ = 42.6 μM) and **7** (IC_50_ = 57.6 μM) showed stronger activities than compounds **2** (IC_50_ = 37.9 μM), **5** (IC_50_ = 154.3 μM) and **8** (IC_50_ = 133.7 μM), respectively, suggesting that the 6′-methyl ester of the glucuronic acid moiety reduced the *α*-glucosidase inhibitory activities [17]. Compared to **6** and **5**, respectively, both **1** and **2** were more potent inhibitors, and the formyl group at C-28 may be responsible for their significant inhibitory properties. Compound **4**, containing the 6-OH group, showed more potent *α*-glucosidase inhibitory activity (IC_50_ = 89.7 μM) than compound **5** (IC_50_ = 154.3 μM), indicating that 6-OH may increase the activity. Interestingly, according to previous literature, compounds **7** and **8** have also shown significant effects on the incensement of serum glucose levels in glucose-loaded rats, especially compound **7**, which strongly inhibited the incensement in serum glucose levels [18].

## 3. Materials and Methods

### 3.1. General Experimental Material

Optical rotations were measured on a Perkin-Elmer 241 polarimeter (Perkin-Elmer, Waltham, MA, USA). IR data were recorded using a Nicolet Magna-IR 750 spectrophotometer (Nicolet Instrument Company, Madison, WI, USA). 1D and 2D NMR spectra were recorded on a Bruker AV-600 spectrometer (600 MHz for ^1^H and 150 MHz for ^13^C, respectively, Bruker Biospin, Fallanden, Switzerland), using tetramethylsilane (TMS) as an internal standard. HR-ESI-MS were measured by an Agilent 6520 Q-TOF LC-MS mass spectrometer (Agilent Technologies, Santa Clara, CA, USA). Analytical TLC were run on silica gel plates (GF_254_, Yantai Institute of Chemical Technology, Yantai, China). Spots were observed under UV light and visualized by spraying with 10% H_2_SO_4_ in ethanol, followed by heating. Column chromatography (CC) was performed on silica gel (200–300 mesh, Qingdao Marine Chemical Factory, Qingdao, China) and Sephadex LH-20 (Amersham Biosciences, Uppsala, Sweden). Acarbose was purchased from Sigma-Aldrich (St. Louis, MO, USA).

### 3.2. Plant Material

The roots of *L. shichuana* were collected in Baoji, Shaanxi Province, People’s Republic of China, in August 2011, and identified by one of the authors (W.-S.W.). A voucher specimen (No. 20110802) was deposited in the herbarium of the College of Life and Environmental Sciences, Minzu University of China, Beijing, People’s Republic of China.

### 3.3. Extraction and Isolation

The chopped, dried roots of *L. shichuana* (1.5 kg) were pulverized and extracted three times with MeOH (each for 7 days) at room temperature. After filtration, the filtrate was concentrated under reduced pressure to yield a residue (135.0 g), and then was suspended in water and partitioned successively with petroleum ether, CHCl_3_, EtOAc, and *n*-butanol, to afford five fractions. The *n*-butanol fraction (15.5 g) was subjected to silica gel CC (500.0 g) eluting with CHCl_3_–MeOH (15:1–0:1, gradient system). On the basis of TLC analysis, seven fractions A-G were obtained. Fraction B (1.1 g) was eluted with CHCl_3_–MeOH (8:1) on CC, further purified by Sephadex LH-20 (MeOH) to afford **2** (16.3 mg). Fraction C (0.9 g) was subjected to CC eluting with CHCl_3_–MeOH (5:1) to afford **3** (2.6 mg) and **5** (9.1 mg). Fraction D (0.5 g) was subjected to CC eluting with CHCl_3_–MeOH (4:1) to afford **4** (20.6 mg). Fraction E (1.3 g) was subjected to CC eluting with CHCl_3_–MeOH (3:1) to afford **1** (9.7 mg) and **7** (7.3 mg). Fraction F (0.7 g) was subjected to Sephadex LH-20 (MeOH) and then was subjected to CC eluting with EtOAc–MeOH (1:1) to afford **6** (5.1 mg). Fraction G (2.4 g) was eluted with CHCl_3_–MeOH (1:1) on CC, further purified by Sephadex LH-20 (MeOH) to afford **8** (7.2 mg).

### 3.4. Spectroscopic Data

*Ligushicoside A* (**1**): Pale yellow, amorphous powder; [α]${}_{D}^{20}$ + 5.1 (*c* = 0.023, MeOH); IR (KBr) *ν*_max_ 3393, 2952, 1715, 1607, 1459, 1033 cm^−1^; ^1^H-NMR (CD_3_OD, 600 MHz) data see Table 1, Figure S1; ^13^C-NMR (CD_3_OD, 150 MHz) data see Table 2, Figure S2; HR-ESI-MS *m*/*z* 633.3998 (calcd. for C_36_H_57_O_9_, 633.4003, Figure S7).

*Ligushicoside B* (**2**): Pale yellow, amorphous powder; [α]${}_{D}^{20}$ + 10.0 (*c* = 0.032, MeOH); IR (KBr) *ν*_max_ 3404, 2943, 1601, 1456, 1027 cm^−1^; ^1^H-NMR (CD_3_OD, 600 MHz) data see Table 1, Figure S8; ^13^C-NMR (CD_3_OD, 150 MHz) data see Table 2, Figure S9; HR-ESI-MS *m*/*z* 669.3972 (calcd. for C_37_H_58_O_9_Na, 669.3979, Figure S13).

*Ligushicoside C* (**3**): Pale yellow, amorphous powder; [α]${}_{D}^{20}$ + 7.8 (*c* = 0.026, MeOH); IR (KBr) *ν*_max_ 3413, 2952, 1604, 1459, 1024 cm^−1^; ^1^H-NMR (CD_3_OD, 600 MHz) data see Table 1, Figure S14; ^13^C-NMR (CD_3_OD, 150 MHz) data see Table 2, Figure S15; HR-ESI-MS *m*/*z* 671.4128 (calcd. for C_37_H_60_O_9_Na, 671.4135, Figure S20).

*Ligushicoside D* (**4**): Pale yellow, amorphous powder; [α]${}_{D}^{20}$ + 8.1 (*c* = 0.031, MeOH); IR (KBr) *ν*_max_ 3403, 2942, 1608, 1461, 1029 cm^−1^; ^1^H-NMR (CD_3_OD, 600 MHz) data see Table 1, Figure S21; ^13^C-NMR (CD_3_OD, 150 MHz) data see Table 2, Figure S22; HR-ESI-MS *m*/*z* 687.4076 (calcd. for C_37_H_60_O_10_Na, 687.4084, Figure S27).

*Ligushicoside E* (**5**): Pale yellow, amorphous powder; [α]${}_{D}^{20}$ + 7.3 (*c* = 0.017, MeOH); IR (KBr) *ν*_max_ 3433, 2952, 1605, 1464, 1032 cm^−1^; ^1^H-NMR (CD_3_OD, 600 MHz) data see Table 1, Figure S28; ^13^C-NMR (CD_3_OD, 150 MHz) data see Table 2, Figure S29; HR-ESI-MS *m*/*z* 671.4128 (calcd. for C_37_H_60_O_9_Na, 671.4135, Figure S33).

### 3.5. Acid Hydrolysis of Saponins

The crude saponin mixture A (compounds **1**–**8**, each 1.0 mg) were treated with 5 N TFA (trifluoroacetic acid, aqueous solution, 3 mL) at 90 °C for 6 h. After extraction with CHCl_3_ (3 × 3 mL), the aqueous layer was neutralized with 0.1 M NaOH and freeze-dried. The sugar was analyzed by TLC (silica gel, CHCl_3_–MeOH–AcOH–H_2_O, 60:32:12:8) for glucuronic acid (*R_f_* 0.30) in comparison with standard sugar. Moreover, the optical rotations of the purified sugar were measured as for d-glucuronic acid, [α]${}_{D}^{20}$ + 8.1 (*c* = 0.26, H_2_O) [12].

### 3.6. α-Glucosidase Inhibitory Activity Assay

*α*-Glucosidase inhibitory activity was examined by the method described by Omar et al. [19]. Acarbose, a definite *α*-glucosidase inhibition, was used as the positive drug.

## 4. Conclusions

In this study, eight oleanane-type saponins, including five new ones, were isolated from the roots of *L. shichuana*. All the structures were established by extensive spectroscopic analysis. The isolates were evaluated on the basis of their *α*-glucosidase inhibitory activity assays. All of the oleanane-type saponins exhibited significant inhibitory activities, with IC_50_ values in the range of 18.7–154.3 μM, which were obviously stronger than the positive control of acarbose (IC_50_ = 190.5 μM). Compounds **1** and **2** showed excellent inhibitory activity against *α*-glucosidase, which might be useful for further developing *α*-glucosidase inhibitors.

## Figures and Tables

**Table 1:** ^1^H-NMR (600 MHz) spectroscopic data for compounds **1**–**5** in CD_3_OD (*δ*_H_, *J* in Hz).

Position	1	2	3	4	5
1a	1.62, m	1.60, m	1.55, m	1.55, m	1.58, m
1b	1.00, m	0.98, m	0.95, m	0.93, m	0.95, m
2a	1.98, m	1.96, m	1.78, m	1.80, m	1.78, m
2b	1.72, m	1.70, m	1.70, m	1.76, m	1.68, m
3	3.19, dd (11.7, 4.3)	3.13, dd (11.8, 4.4)	3.07, dd (9.9, 5.7)	3.07, dd (9.9, 5.7)	3.14, dd (11.2, 4.6)
5	0.81, m	0.77, m	0.75, m	0.75, m	0.76, m
6a	1.59, m	1.57, m	4.51, br s	4.51, br s	1.57, m
6b	1.45, m	1.43, m			1.43, m
7a	1.43, m	1.41, m	1.72, m	1.73, m	1.55, m
7b	1.27, m	1.25, m	1.55, m	1.58, m	1.36, m
9	1.58, m	1.56, m	1.58, m	1.58, m	1.54, m
11a	1.93, m	1.92, m	2.03, m	2.02, m	1.92, m
11b	1.92, m	1.91, m	1.91, m	1.90, m	1.85, m
12	5.34, t-like (1.5)	5.31, t-like (1.5)	5.27, br s	5.27, br s	5.34, br s
15a	1.82, m	1.79, m	1.80, m	1.40, m	1.40, m
15b	1.53, m	1.51, m	1.25, m	1.20, m	1.21, m
16	4.32, dd (11.9, 4.5)	4.29, dd (12.0, 4.6)	4.13, dd (11.4, 4.5)	4.24, dd (11.7, 4.8)	4.24, dd (12.0, 4.8)
18	2.78, dd (14.0, 4.2)	2.76, dd (14.0, 4.5)	2.18, m	2.21, m	2.18, m
19a	1.70, m	1.67, m	1.73, m	1.72, m	1.71, m
19b	1.19, m	1.16, m	1.03, m	1.06, m	1.02, m
21a	1.58, m	1.56, m	1.90, m	1.41, m	1.41, m
21b	1.37, m	1.34, m	1.15, m	1.21, m	1.21, m
22a	2.09, m	2.06, m	1.39, m	2.21, m	2.10, m
22b	1.26, m	1.23, m	1.11, m	1.41, m	1.40, m
23	1.08, s	1.05, s	1.13, s	1.13, s	1.06, s
24	0.88, s	0.85, s	1.23, s	1.23, s	0.86, s
25	0.98, s	0.95, s	1.33, s	1.33, s	0.97, s
26	0.83, s	0.81, s	1.28, s	1.30, s	1.03, s
27	1.25, s	1.23, s	1.19, s	1.20, s	1.24, s
28a	9.76, s	9.73, s	0.79, s	3.80, d (9.7)	3.81, d (9.7)
28b				3.26, m	3.27, m
29	0.96, s	0.93, s	0.89, s	0.90, s	0.90, s
30	0.96, s	0.94, s	0.92, s	0.93, s	0.92, s
GluA-1′	4.38, d (8.2)	4.37, d (7.8)	4.37, d (7.7)	4.37, d (7.8)	4.40, d (7.5)
2′	3.24, t (8.3)	3.22, t (8.5)	3.24, t (8.4)	3.24, t (8.4)	3.26, t (8.4)
3′	3.39, t (9.2)	3.34, t (9.1)	3.35, t (9.1)	3.35, t (9.1)	3.37, t (9.0)
4′	3.47, t (8.8)	3.49, t (9.4)	3.50, t (9.4)	3.49, t (9.4)	3.53, t (9.2)
5′	3.62, d (10.0)	3.81, d (9.8)	3.80, d (9.8)	3.80, d (9.7)	3.83, br d (10.0)
6′-OCH3		3.77, s	3.76, s	3.76, s	3.79, s

**Table 2:** ^13^C-NMR (150 MHz) spectroscopic data for compounds **1**–**5** in CD_3_OD (*δ*_C_, type).

Position	1	2	3	4	5
1	38.4, CH_2_	38.3, CH_2_	42.1, CH_2_	42.1, CH_2_	39.9, CH_2_
2	25.5, CH_2_	25.6, CH_2_	27.3, CH_2_	27.3, CH_2_	27.0, CH_2_
3	89.4, CH	89.6, CH	91.3, CH	91.3, CH	91.1, CH
4	38.8, C	38.8, C	41.2, C	41.2, C	41.6, C
5	55.6, CH	55.5, CH	57.3, CH	57.3, CH	57.0, CH
6	17.9, CH_2_	17.8, CH_2_	68.6, CH	68.6, CH	19.3, CH_2_
7	32.6, CH_2_	32.6, CH_2_	41.6, CH_2_	41.7, CH_2_	33.7, CH_2_
8	39.5, C	39.4, C	40.4, C	40.4, C	40.9, C
9	46.8, CH	46.7, CH	48.7, CH	48.6, CH	48.3, CH
10	36.4, C	36.4, C	37.4, C	36.7, C	36.7, C
11	23.1, CH_2_	23.1, CH_2_	24.7, CH_2_	24.7, CH_2_	24.1, CH_2_
12	123.1, CH	123.0, CH	123.8, CH	124.3, CH	124.0, CH
13	142.0, C	141.9, C	144.5, C	143.6, C	144.3, C
14	43.4, C	43.4, C	45.4, C	45.1, C	44.7, C
15	36.5, CH_2_	36.5, CH_2_	36.4, CH_2_	34.9, CH_2_	35.0, CH_2_
16	63.8, CH	63.8, CH	66.4, CH	68.0, CH	67.8, CH
17	52.8, C	52.7, C	38.6, C	41.5, C	40.2, C
18	41.8, CH	41.7, CH	50.8, CH	45.1, CH	45.1, CH
19	45.7, CH_2_	45.8, CH_2_	48.1, CH_2_	47.9, CH_2_	47.9, CH_2_
20	30.0, C	30.0, C	31.9, C	30.8, C	31.7, C
21	32.6, CH_2_	32.6, CH_2_	35.4, CH_2_	34.9, CH_2_	34.8, CH_2_
22	22.3, CH_2_	22.3, CH_2_	31.7, CH_2_	26.0, CH_2_	26.0, CH_2_
23	27.1, CH_3_	27.0, CH_3_	28.3, CH_3_	28.2, CH_3_	28.5, CH_3_
24	15.6, CH_3_	15.5, CH_3_	18.5, CH_3_	18.5, CH_3_	17.0, CH_3_
25	14.6, CH_3_	14.6, CH_3_	17.6, CH_3_	17.5, CH_3_	16.2, CH_3_
26	16.3, CH_3_	16.2, CH_3_	18.9, CH_3_	18.6, CH_3_	17.7, CH_3_
27	25.6, CH_3_	25.5, CH_3_	27.8, CH_3_	27.5, CH_3_	27.0, CH_3_
28	208.0, CH	208.0, CH	22.4, CH_3_	69.0, CH_2_	69.0, CH_2_
29	32.0, CH_3_	32.0, CH_3_	33.9, CH_3_	31.8, CH_3_	33.7, CH_3_
30	22.6, CH_3_	22.6, CH_3_	24.5, CH_3_	24.3, CH_3_	24.3, CH_3_
GluA-1′	105.3, CH	105.6, CH	107.1, CH	107.1, CH	107.1, CH
2′	75.3, CH	73.9, CH	75.4, CH	75.4, CH	75.4, CH
3′	78.1, CH	78.1, CH	77.6, CH	77.6, CH	77.6, CH
4′	74.1, CH	71.8, CH	73.3, CH	73.3, CH	73.3, CH
5′	76.6, CH	76.1, CH	76.7, CH	76.7, CH	76.7, CH
6′	175.5, C	170.0, C	171.5, C	171.5, C	171.5, C
6′-OCH_3_		51.4, CH_3_	52.9, CH_3_	52.8, CH_3_	52.8, CH_3_

**Table 3:** Inhibitory effects of compounds **1**–**8** and acarbose against *α*-glucosidase.

Compounds	IC_50_ (μM)	Compounds	IC_50_ (μM)
**1**	18.7 ± 1.7	**5**	154.3 ± 11.2
**2**	37.9 ± 3.6	**6**	42.6 ± 5.9
**3**	104.9 ± 4.3	**7**	57.6 ± 6.8
**4**	89.7 ± 5.1	**8**	133.7 ± 3.3
Acarbose	190.5 ± 3.1		

## Supplementary Materials

Supplementary data (Figures S1–S33) associated with this article, including HRESIMS, 1D and 2D NMR spectra data (**1**–**5**) can be found in the online version.
