# Supplementary material for: Oleanane-Type Saponins from the Roots of Ligulariopsis shichuana and Their α-Glucosidase Inhibitory Activities
Source: Molecules. 2017 Nov 17;22(11):1981. doi: 10.3390/molecules22111981 (PMC6150216; doi:10.3390/molecules22111981)
Supplement: Supplementary file 1 [file molecules-22-01981-s001.pdf]

Supporting Information

Table of Contents

	Table of Contents	Page
Figure S1.	^{1}H NMR (600 MHz, CD_3OD) spectrum of compound **1**	4
Figure S2.	^{13}C NMR (150 MHz, CD_3OD) spectrum of compound **1**	5
Figure S3.	^{1}H-^{1}H COSY spectrum (CD_3OD) of compound **1**	6
Figure S4.	HSQC spectrum (CD_3OD) of compound **1**	7
Figure S5.	HMBC spectrum (CD_3OD) of compound **1**	8
Figure S6.	NOESY spectrum (CD_3OD) of compound **1**	9
Figure S7.	HRESIMS spectrum of compound **1**	10
Figure S8.	^{1}H NMR (600 MHz, CD_3OD) spectrum of compound **2**	11
Figure S9.	^{13}C NMR (150 MHz, CD_3OD) spectrum of compound **2**	12
Figure S10.	^{1}H-^{1}H COSY spectrum (CD_3OD) of compound **2**	13
Figure S11.	HSQC spectrum (CD_3OD) of compound **2**	14
Figure S12.	HMBC spectrum (CD_3OD) of compound **2**	15
Figure S13.	HRESIMS spectrum of compound **2**	16
Figure S14.	^{1}H NMR (600 MHz, CD_3OD) spectrum of compound **3**	17
Figure S15.	^{13}C NMR (150 MHz, CD_3OD) spectrum of compound **3**	18
Figure S16.	^{1}H-^{1}H COSY spectrum (CD_3OD) of compound **3**	19
Figure S17.	HSQC spectrum (CD_3OD) of compound **3**	20
Figure S18.	HMBC spectrum (CD_3OD) of compound **3**	21
Figure S19.	NOESY spectrum (CD_3OD) of compound **3**	22
Figure S20.	HRESIMS spectrum of compound **3**	23
Figure S21.	^{1}H NMR (600 MHz, CD_3OD) spectrum of compound **4**	24
Figure S22.	^{13}C NMR (150 MHz, CD_3OD) spectrum of compound **4**	25
Figure S23.	^{1}H-^{1}H COSY spectrum (CD_3OD) of compound **4**	26
Figure S24.	HSQC spectrum (CD_3OD) of compound **4**	27
Figure S25.	HMBC spectrum (CD_3OD) of compound **4**	28
Figure S26.	NOESY spectrum (CD_3OD) of compound **4**	29
Figure S27.	HRESIMS spectrum of compound **4**	30
Figure S28.	^{1}H NMR (600 MHz, CD_3OD) spectrum of compound **5**	31
Figure S29.	^{13}C NMR (150 MHz, CD_3OD) spectrum of compound **5**	32
Figure S30.	^{1}H-^{1}H COSY spectrum (CD_3OD) of compound **5**	33
Figure S31.	HSQC spectrum (CD_3OD) of compound **5**	34
Figure S32.	HMBC spectrum (CD_3OD) of compound **5**	35
Figure S33.	HRESIMS spectrum of compound **5**	36

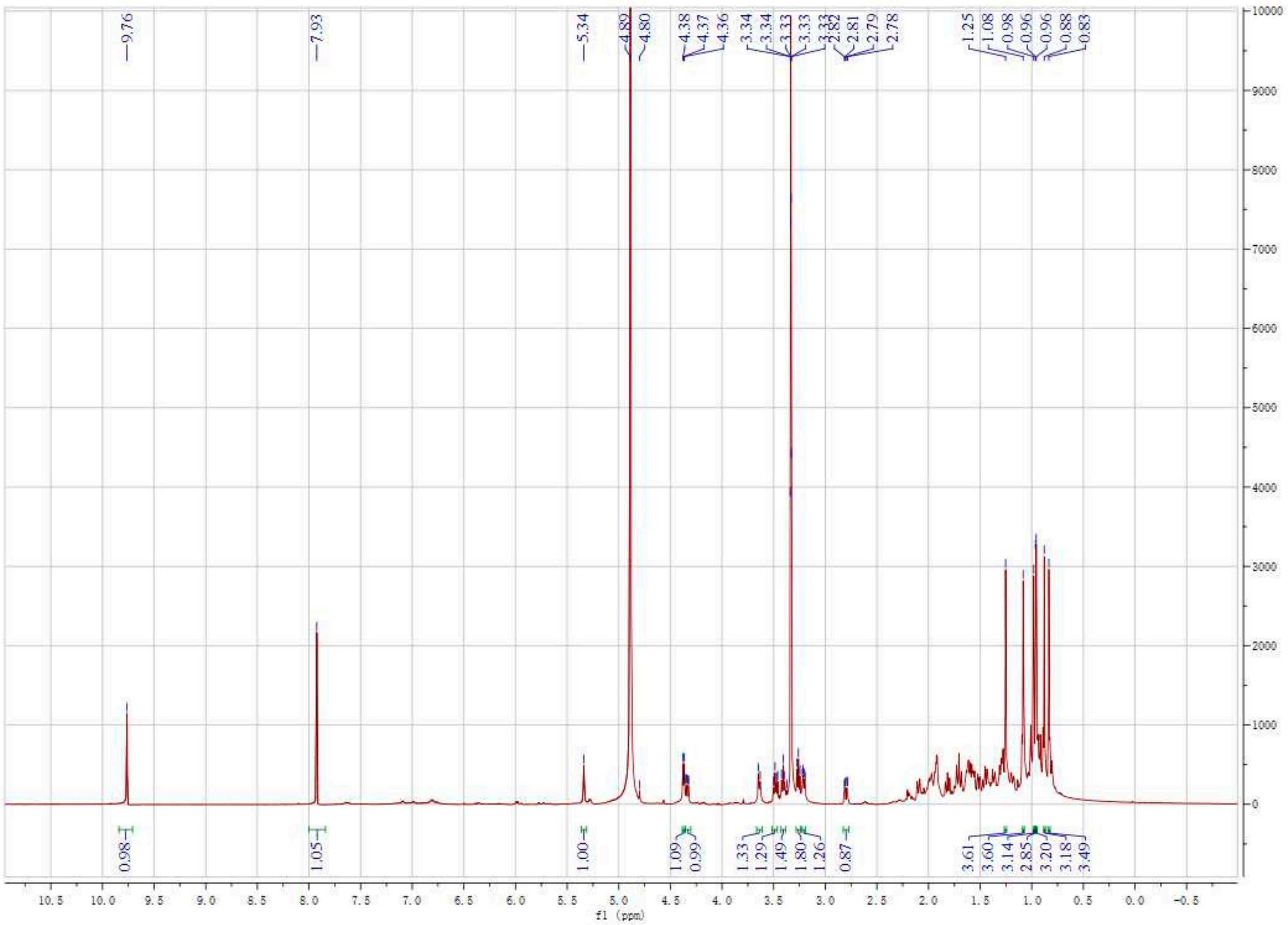

Figure S1. ^{1}H NMR (600 MHz, CD_3OD) spectrum of compound **1**

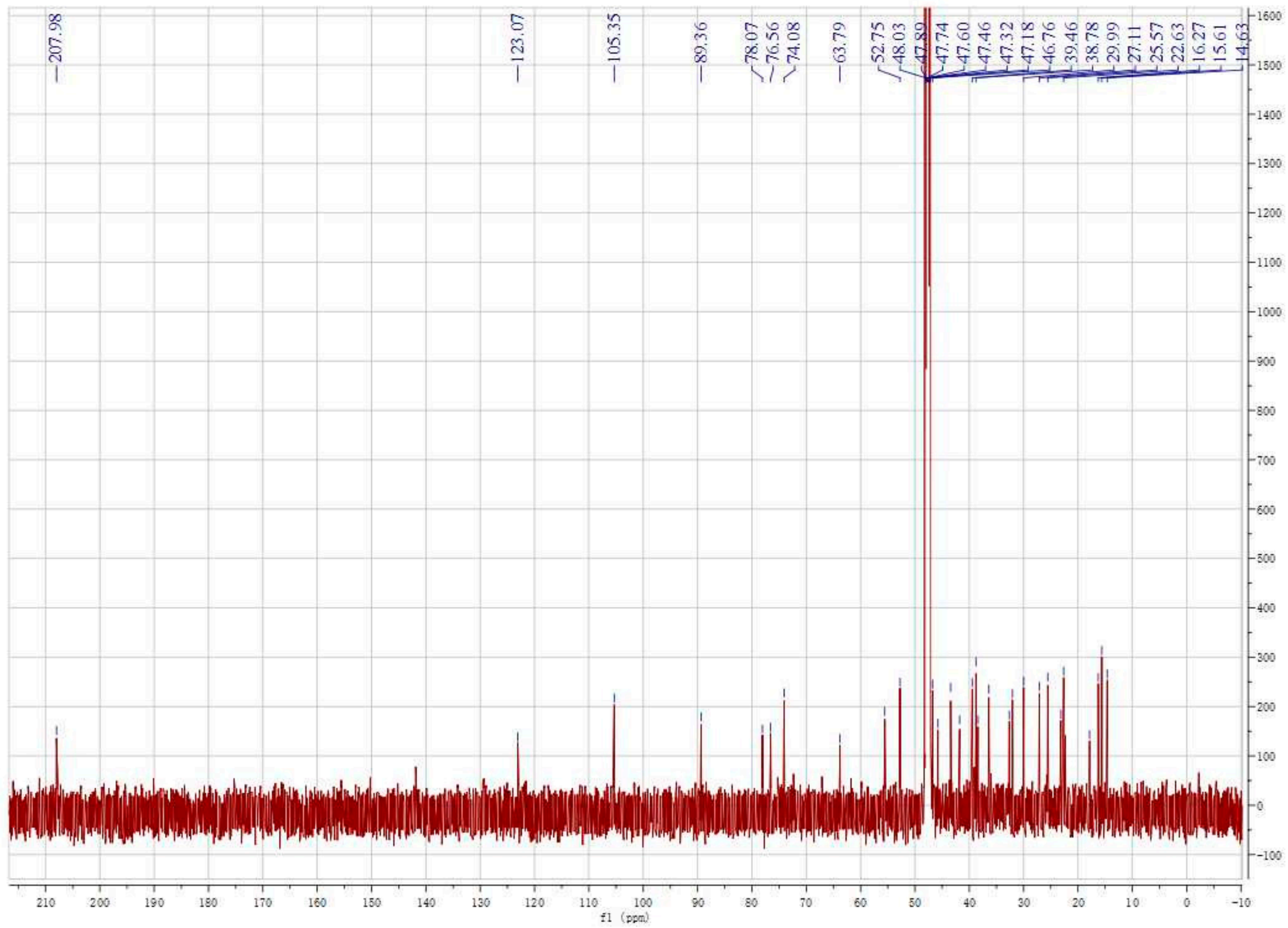

Figure S2. ^{13}C-NMR (150 MHz, CD_3OD) spectrum of compound **1**

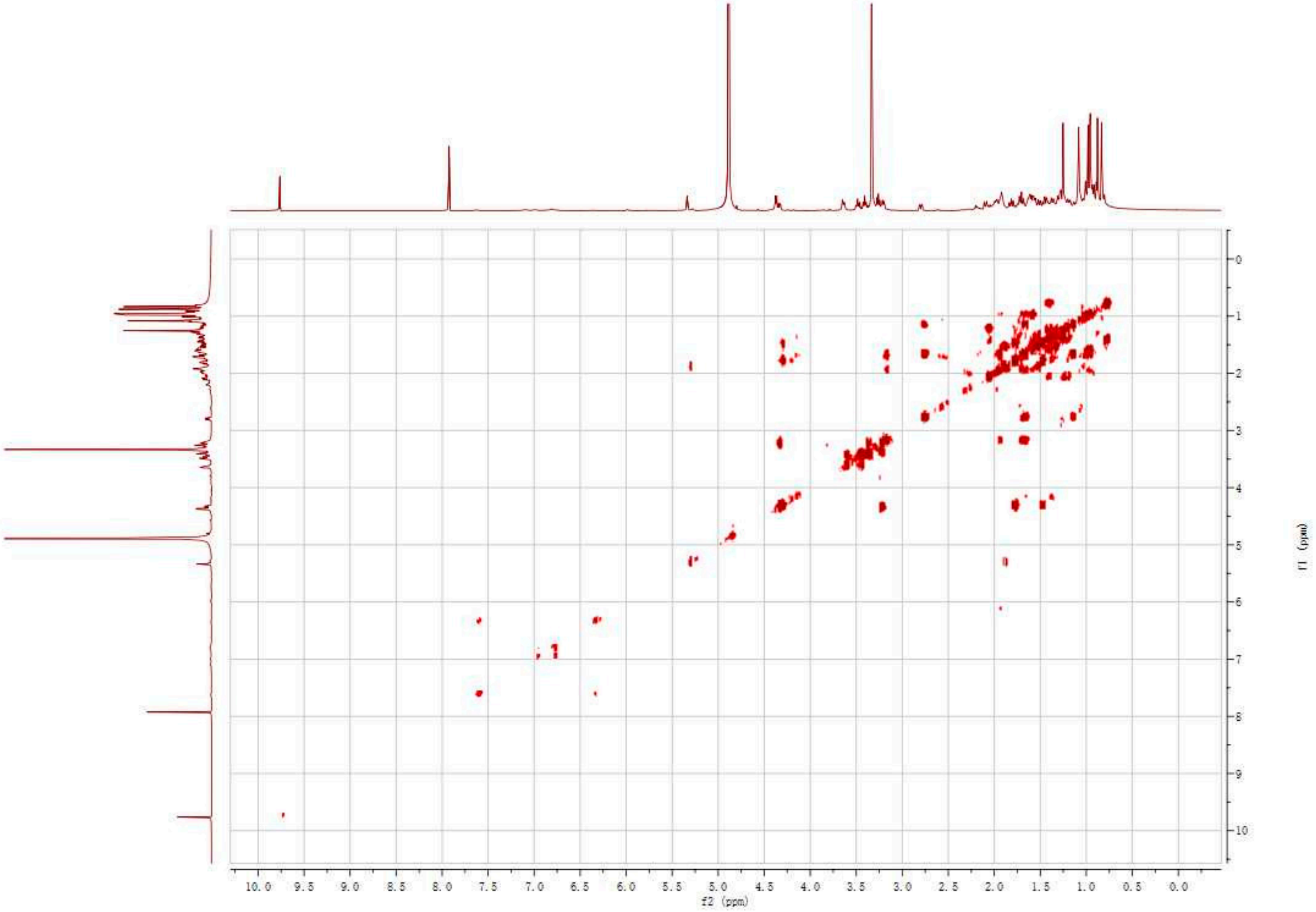

Figure S3. ^{1}H-^{1}H COSY spectrum (CD_3OD) of compound **1**

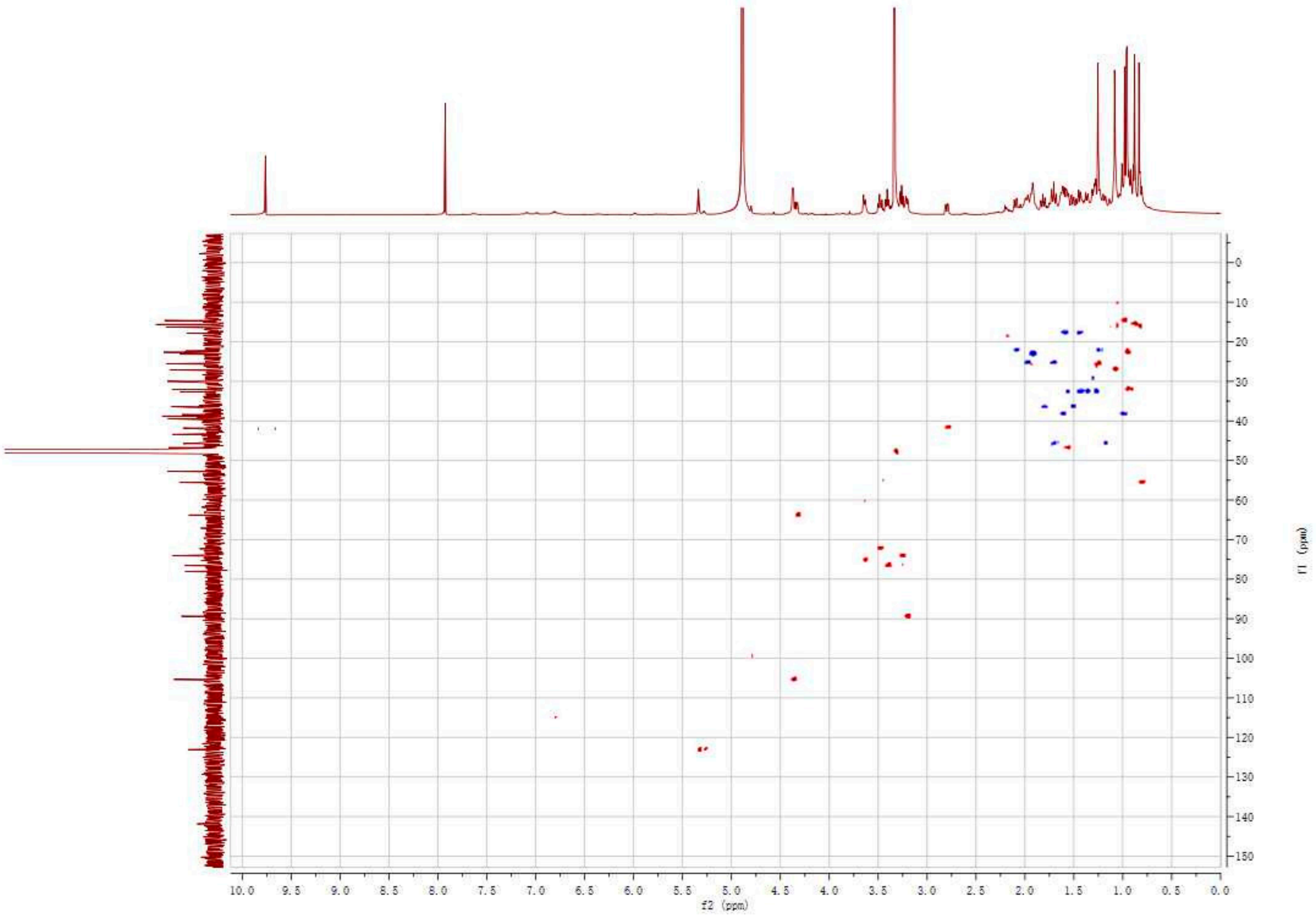

Figure S4. HSQC spectrum (CD_3OD) of compound **1**

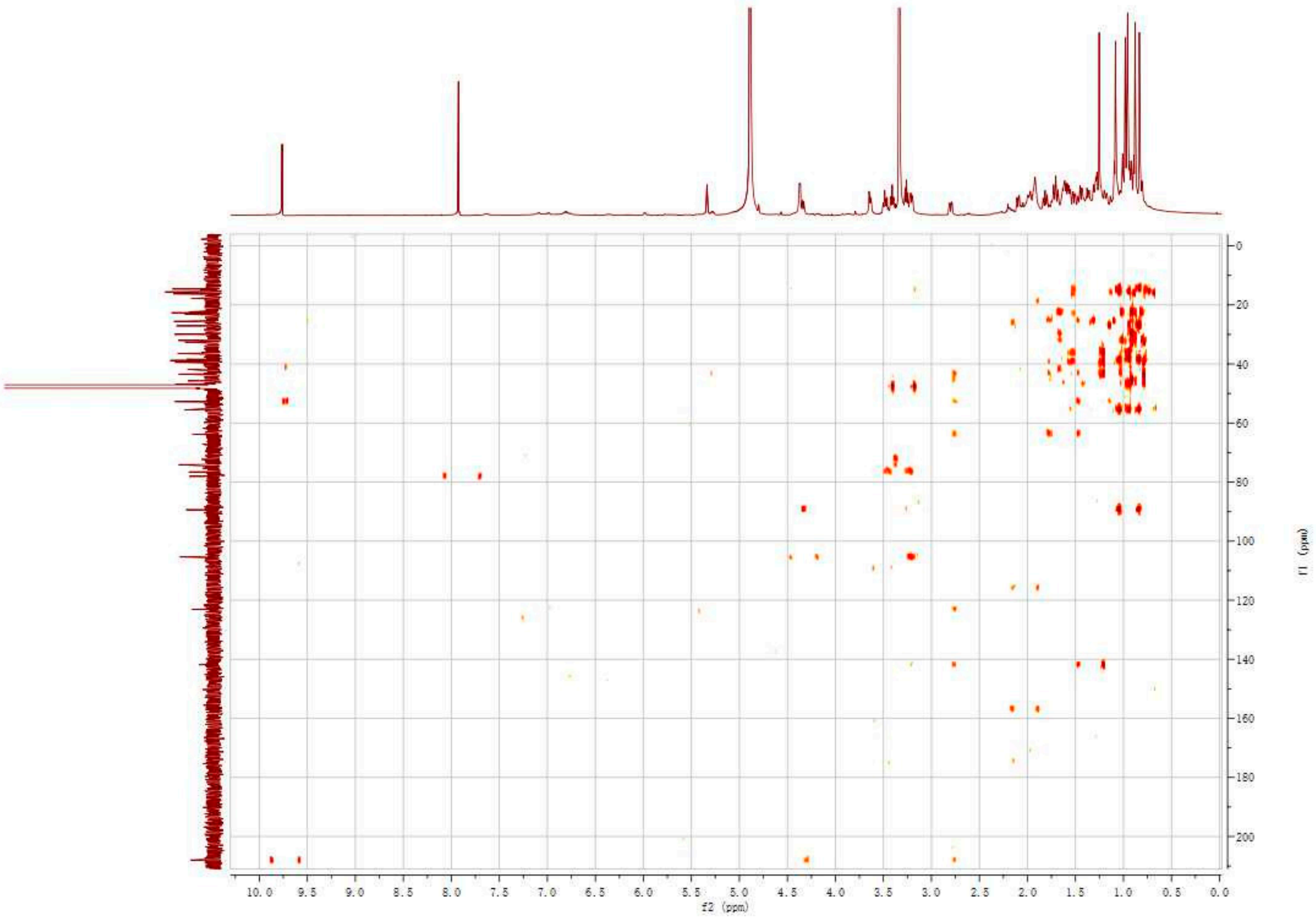

Figure S5. HMBC spectrum (CD_3OD) of compound **1**

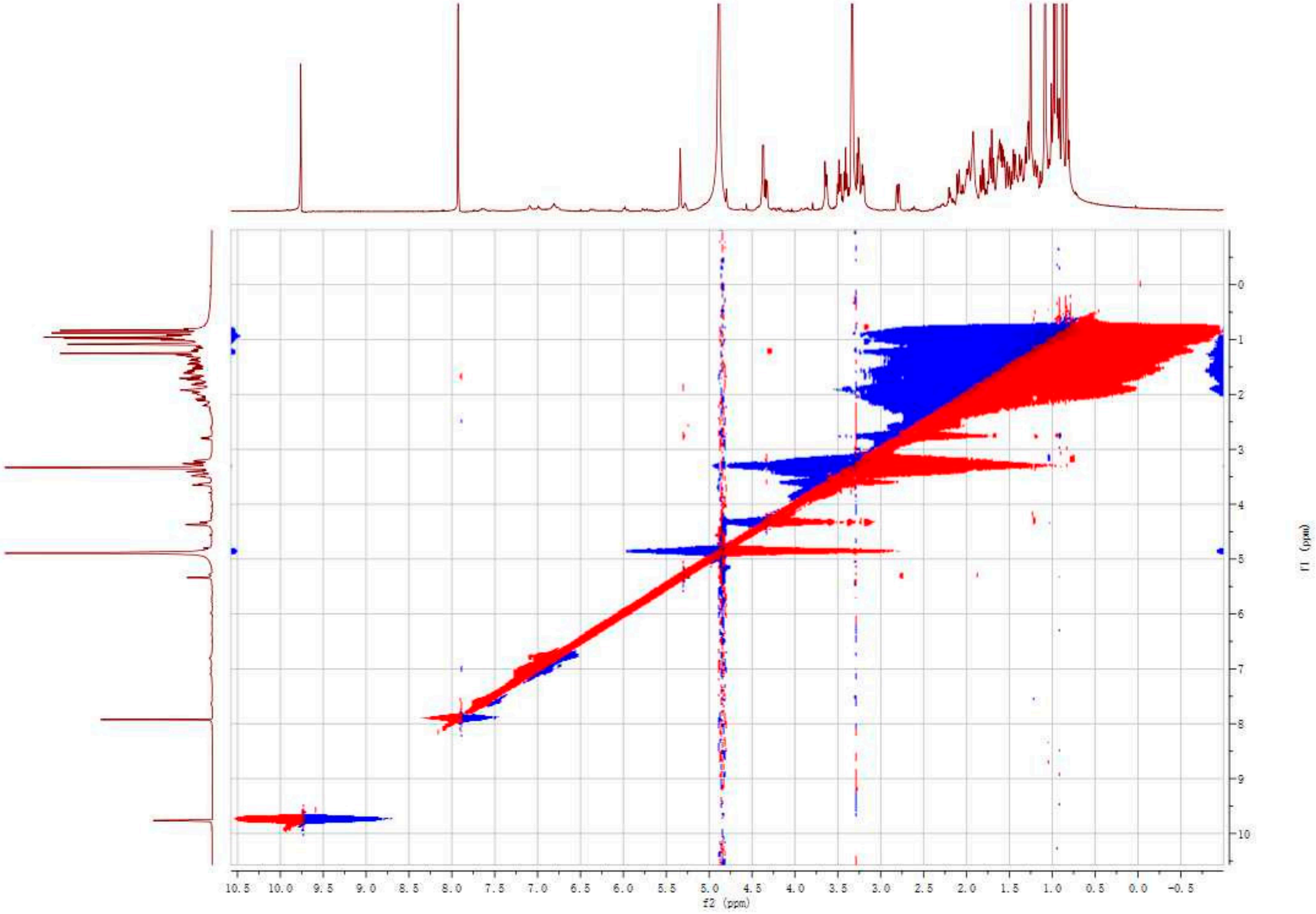

Figure S6. NOESY spectrum (CD_3OD) of compound **1**

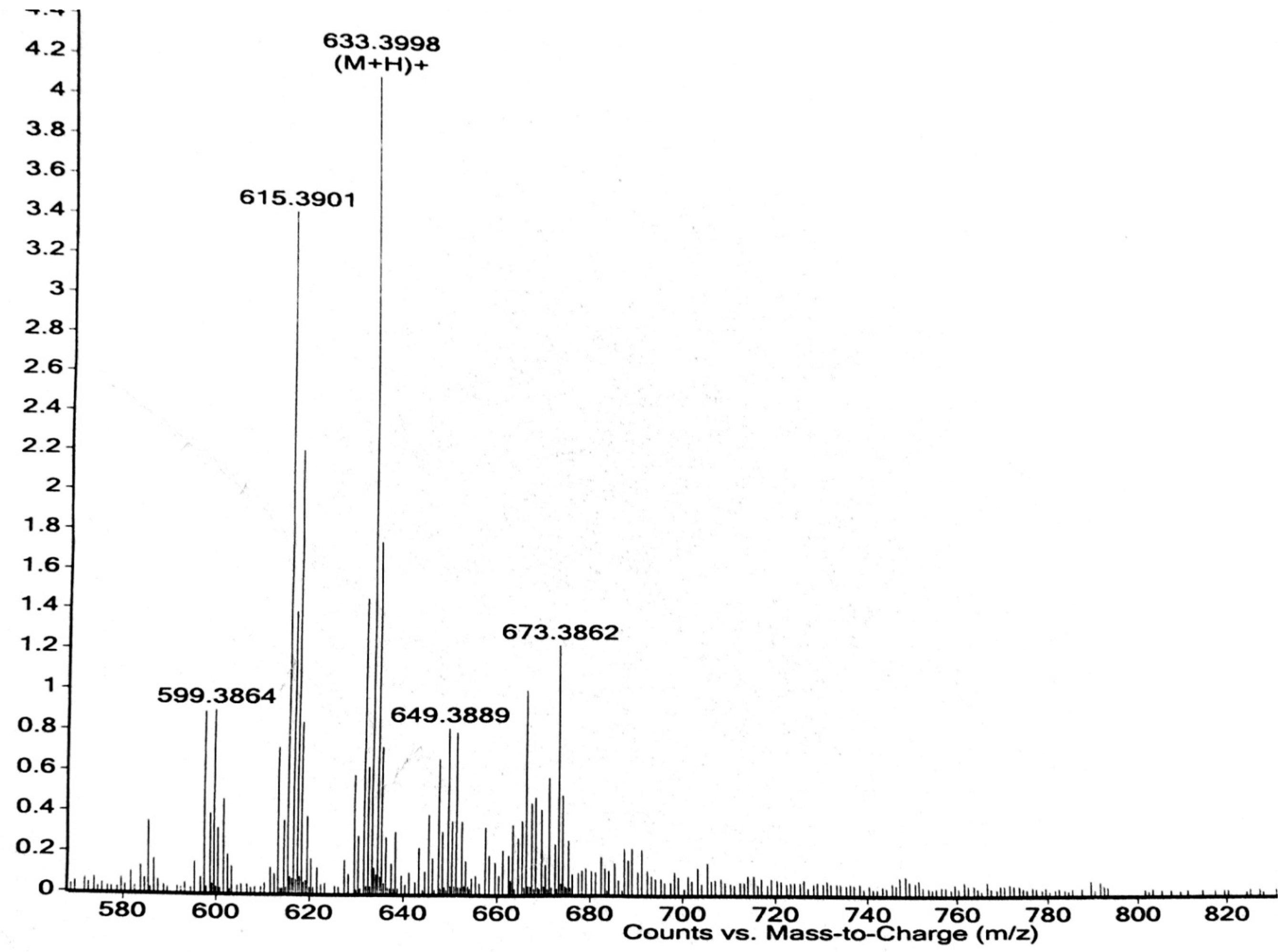

Figure S7. HRESIMS spectrum of compound **1**

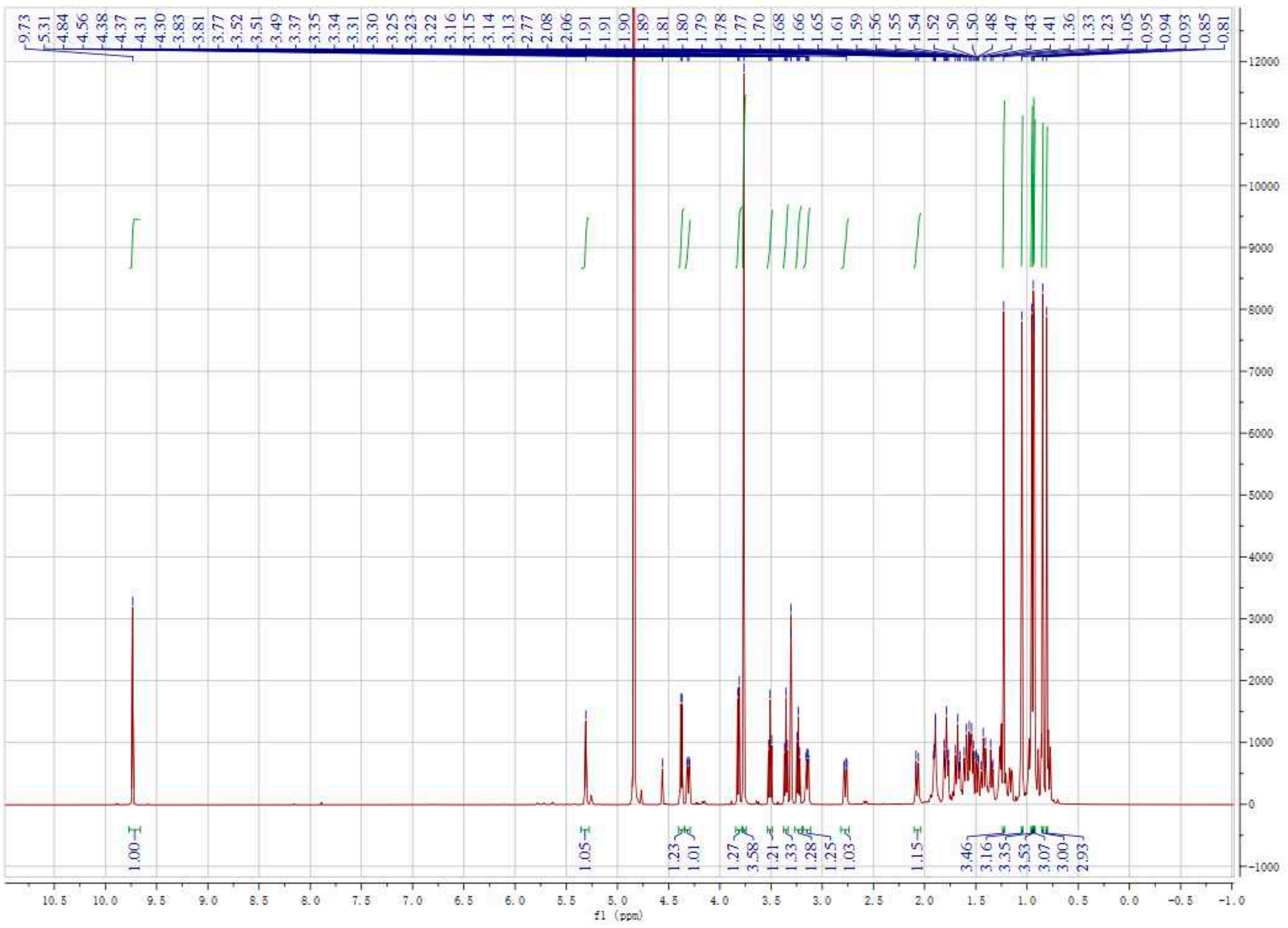

Figure S8. ^{1}H NMR (600 MHz, CD_3OD) spectrum of compound **2**

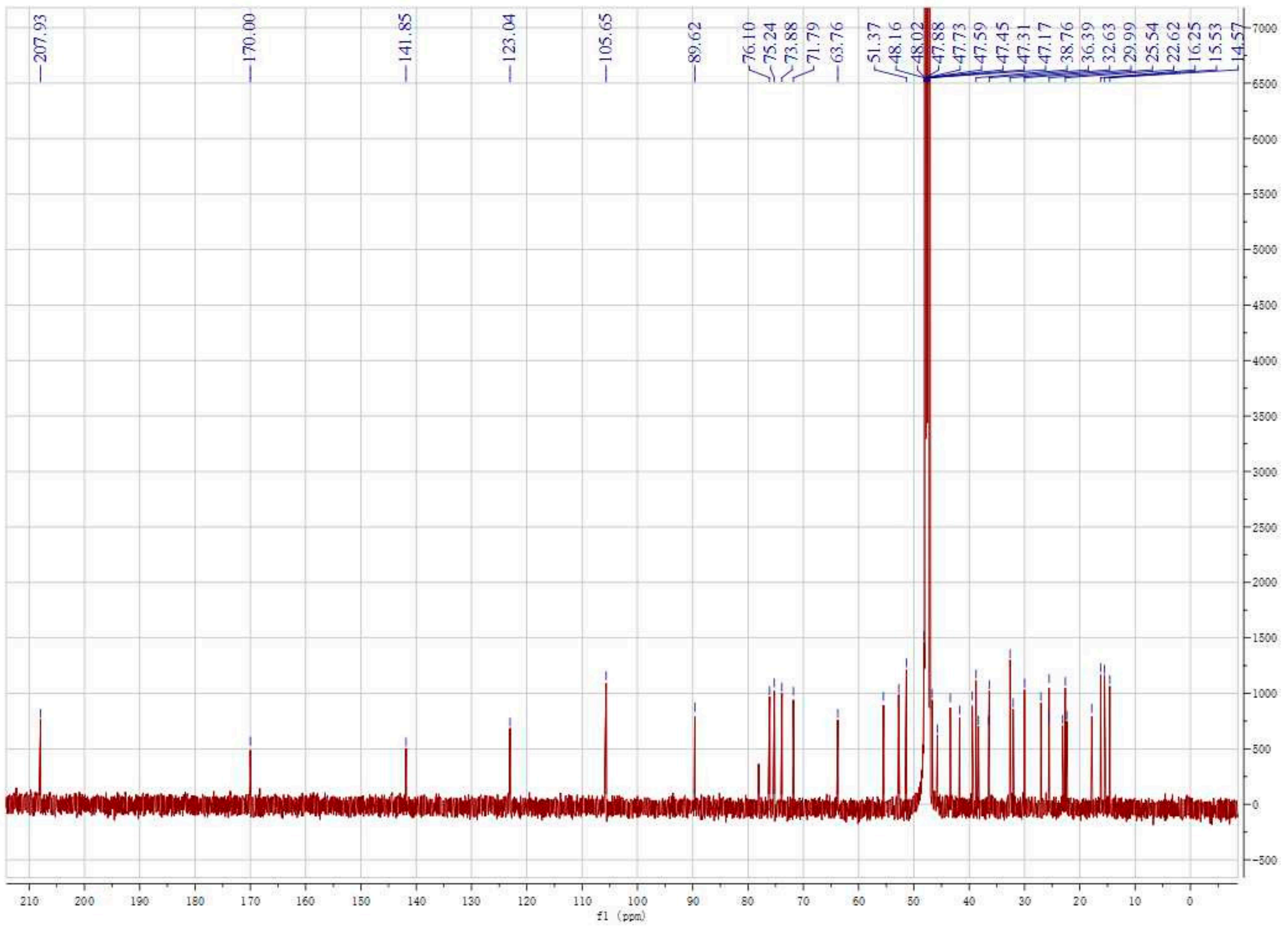

Figure S9. ^{13}C NMR (150 MHz, CD_3OD) spectrum of compound **2**

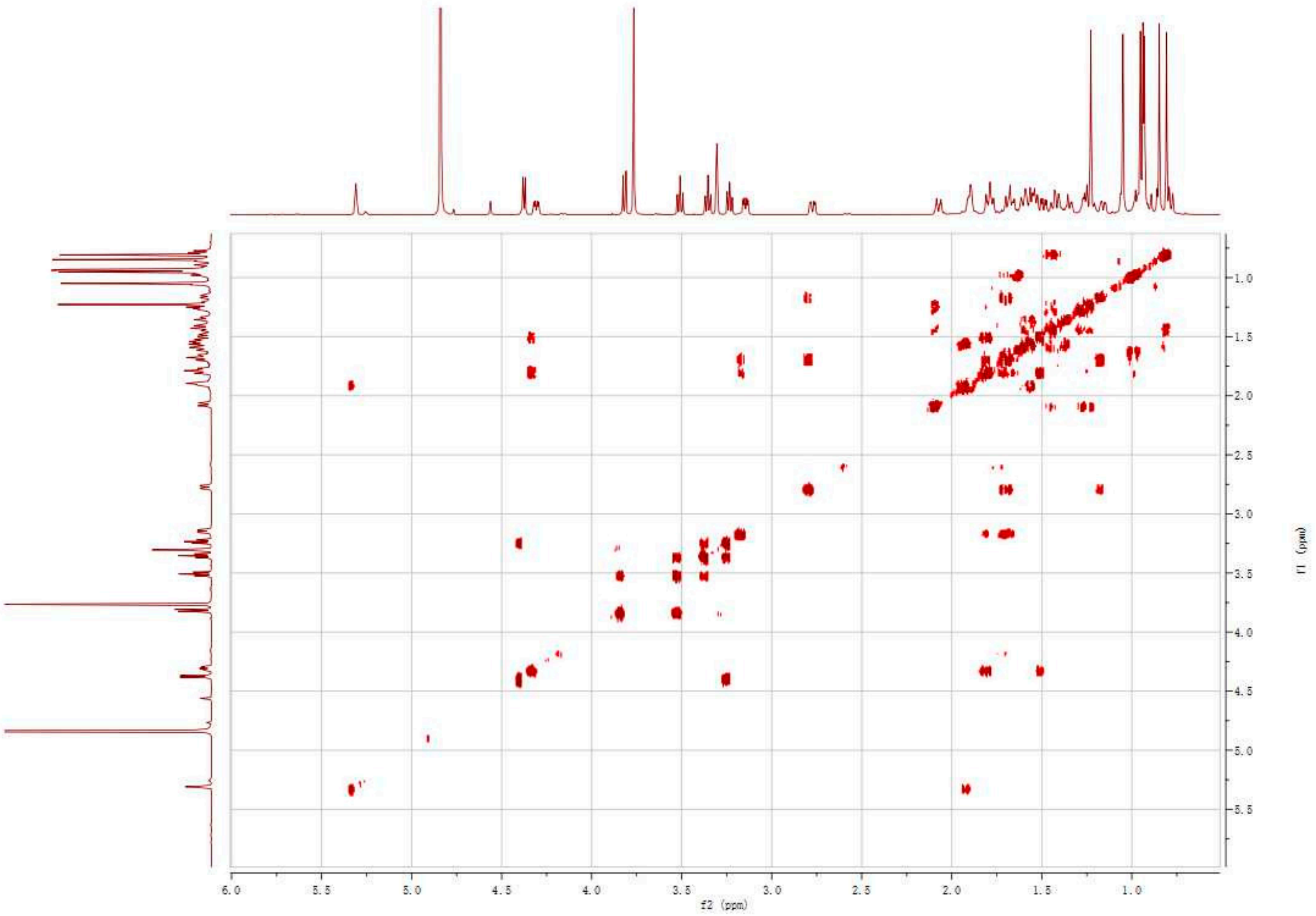

Figure S10. ^{1}H-^{1}H COSY spectrum (CD_3OD) of compound **2**

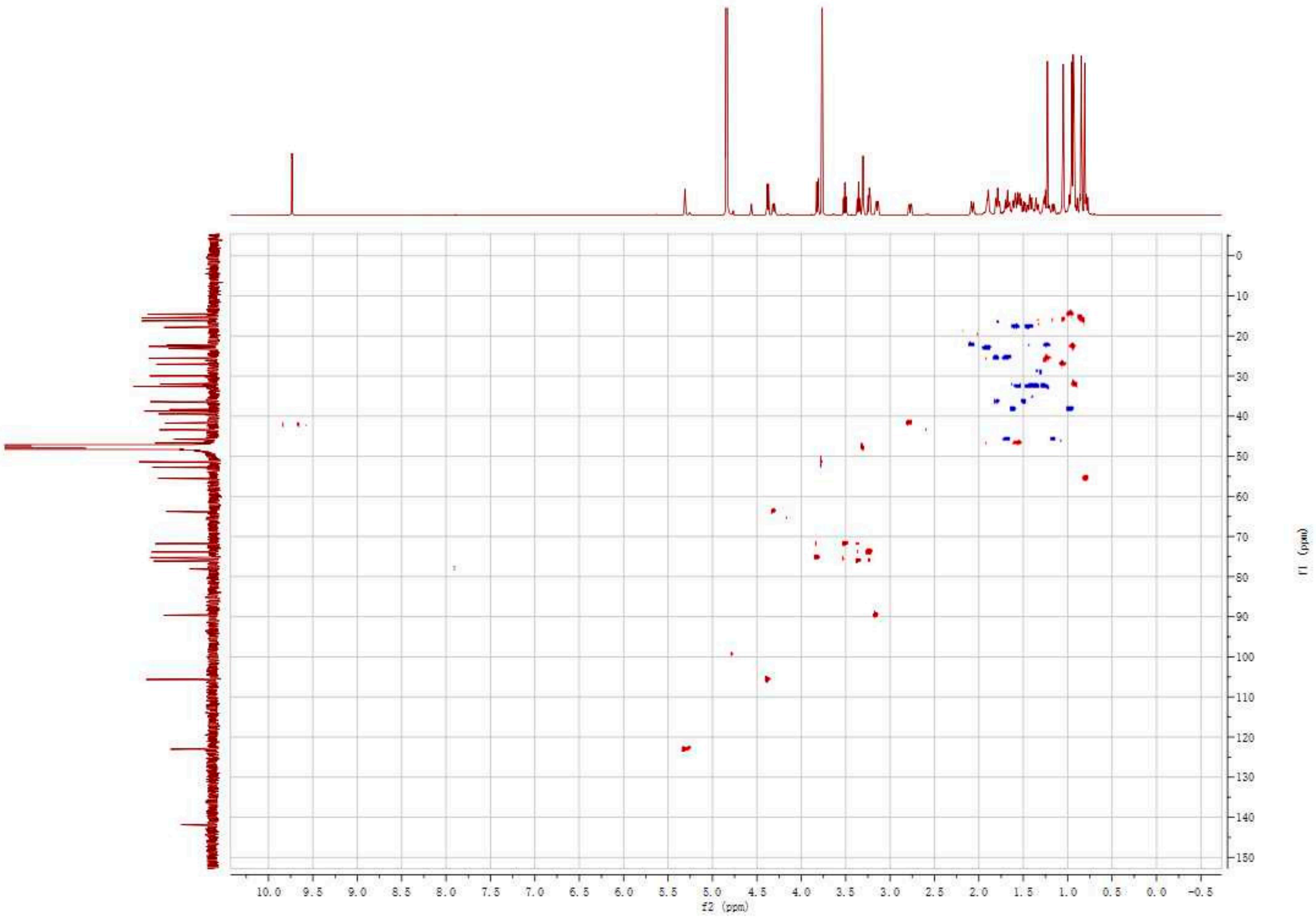

Figure S11. HSQC spectrum (CD_3OD) of compound **2**

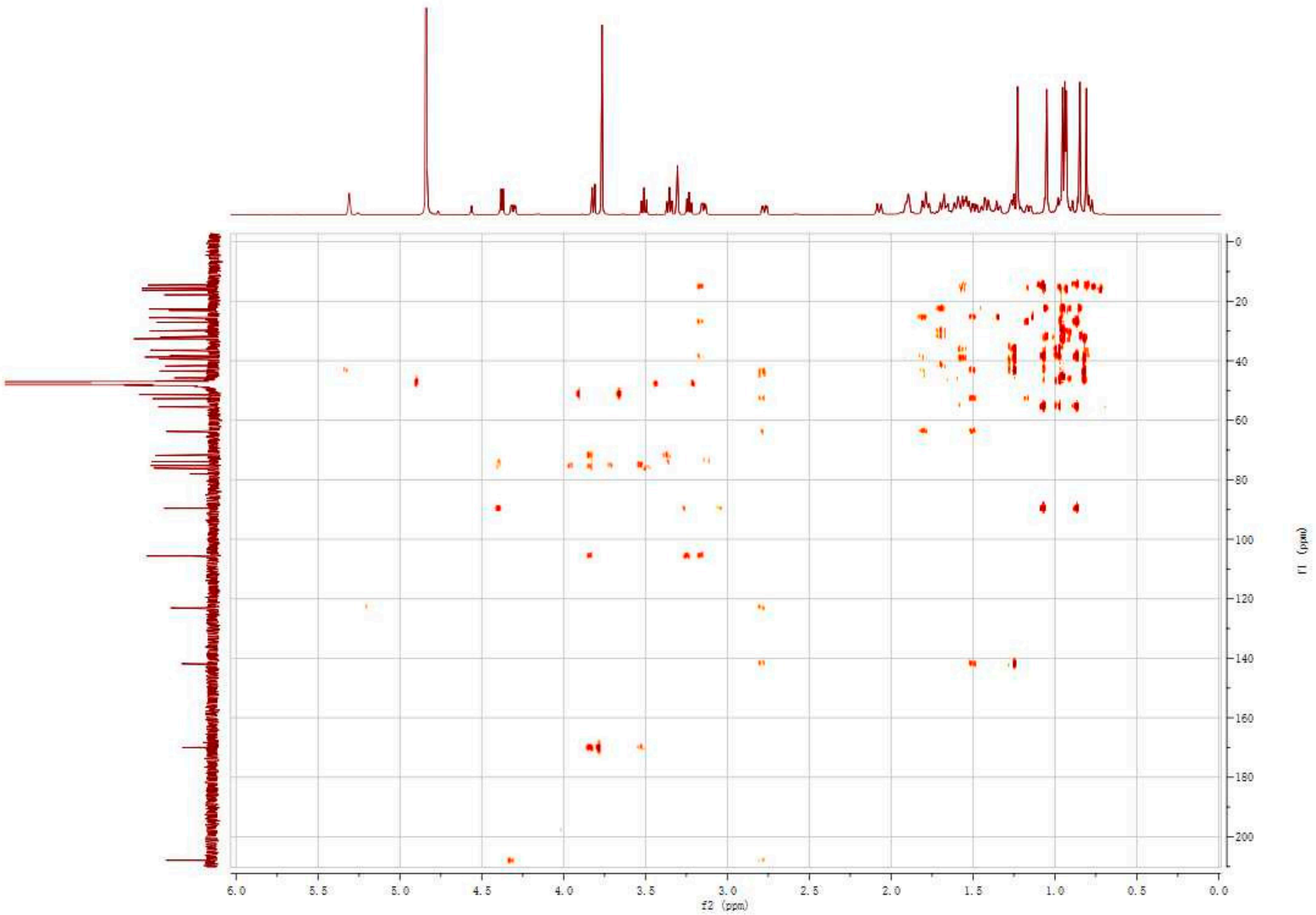

Figure S12. HMBC spectrum (CD_3OD) of compound **2**

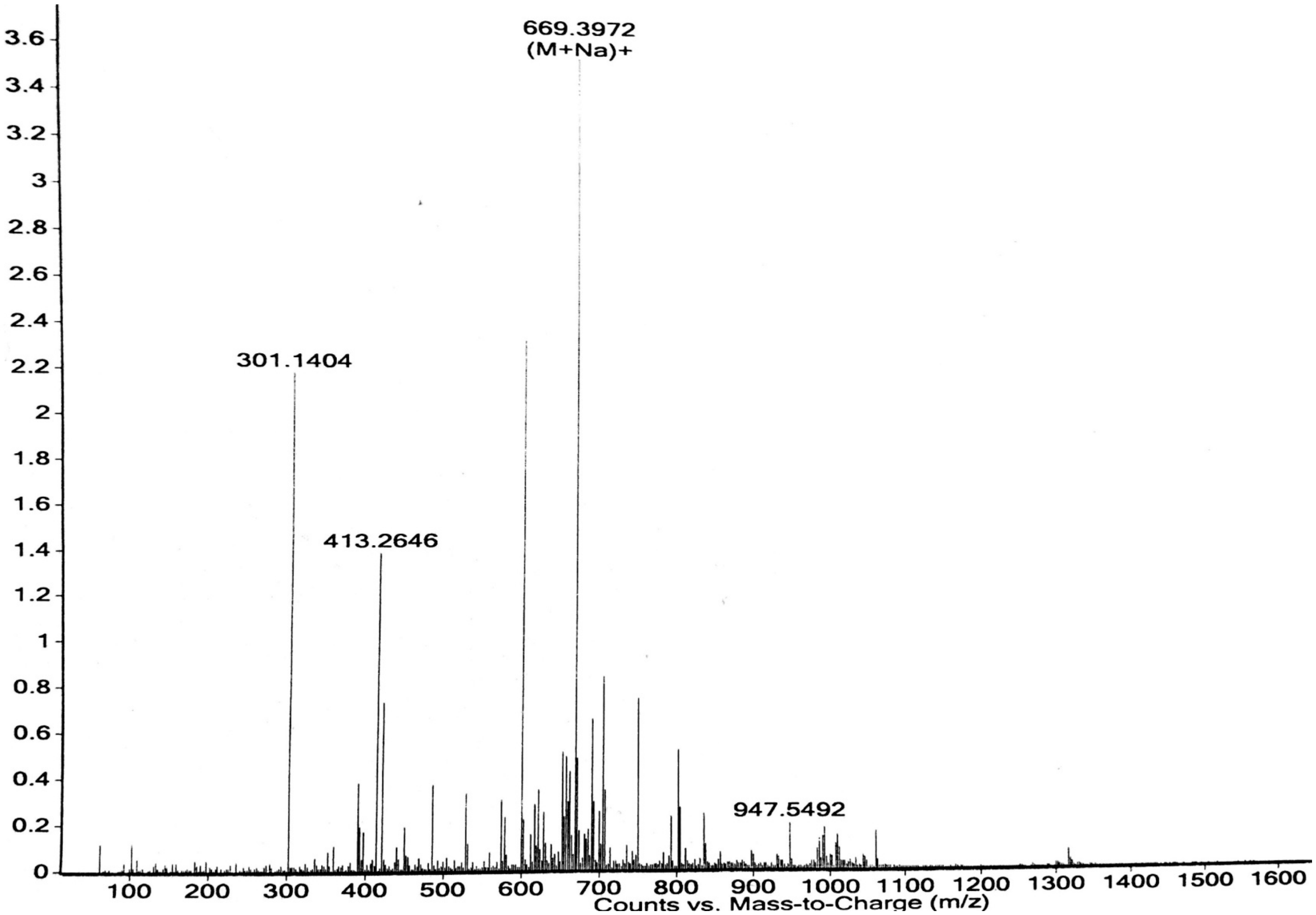

Figure S13. HRESIMS spectrum of compound **2**

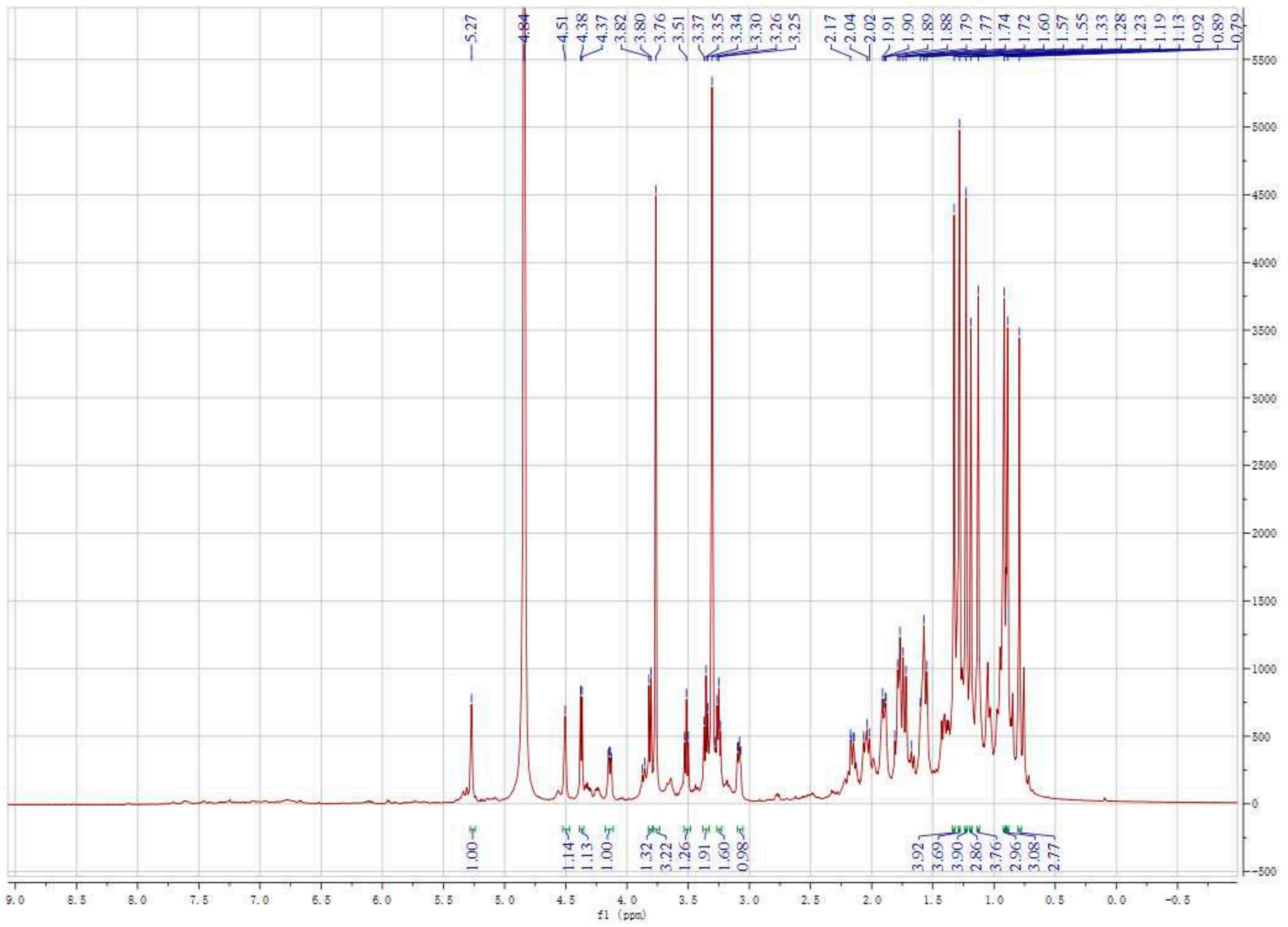

Figure S14. ^{1}H NMR (600 MHz, CD_3OD) spectrum of compound **3**

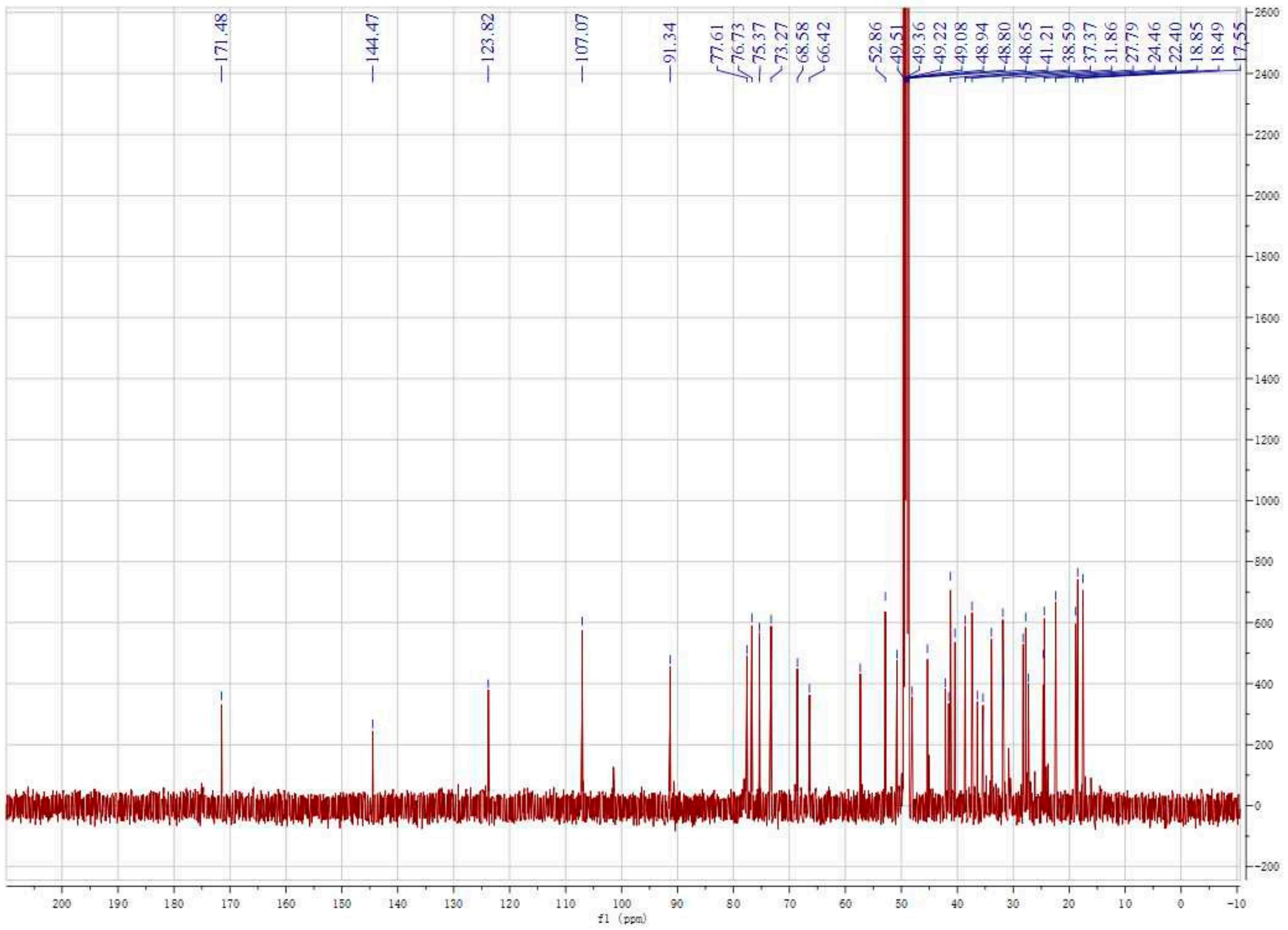

Figure S15. ^{13}C NMR (150 MHz, CD_3OD) spectrum of compound **3**

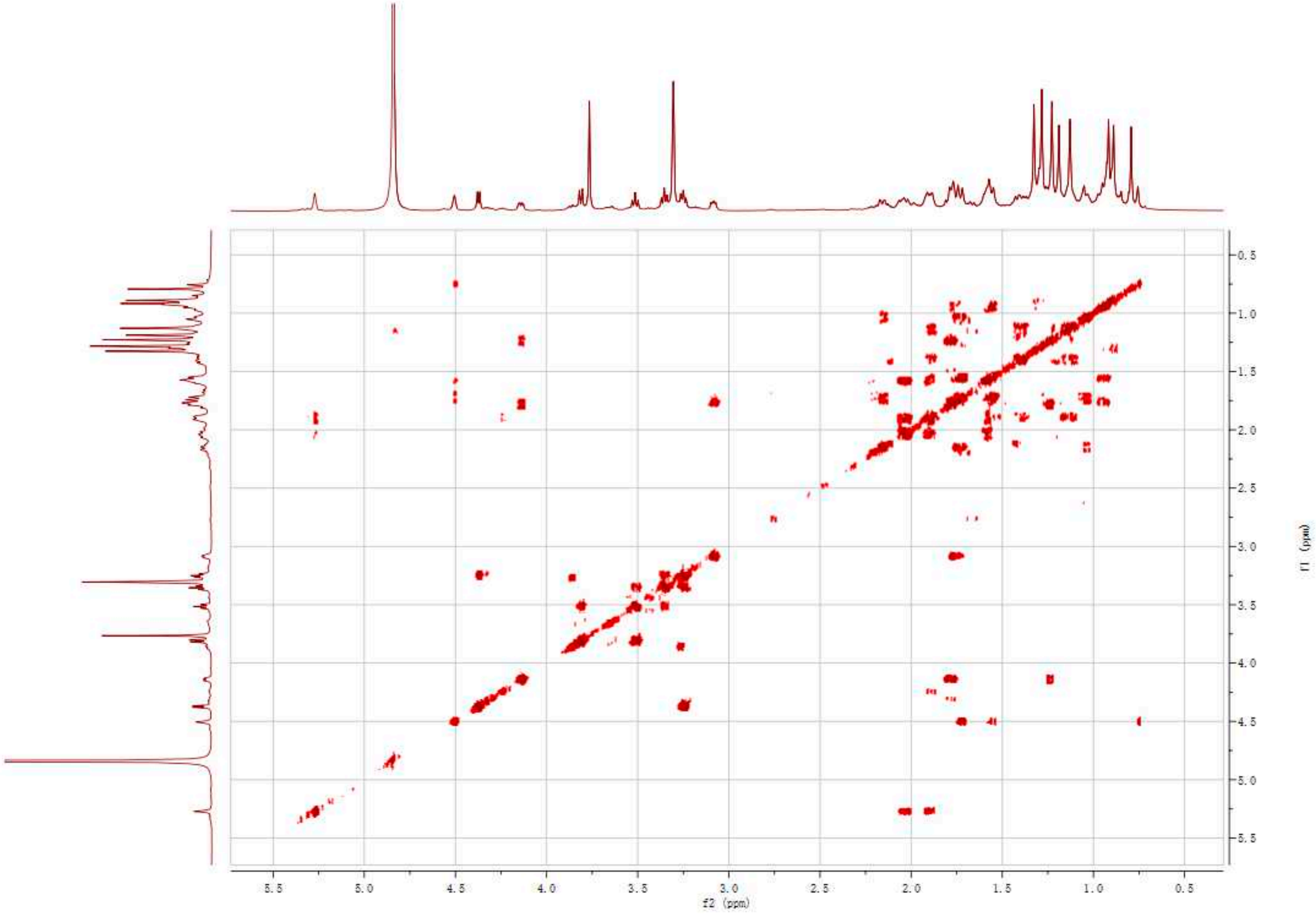

Figure S16. ^{1}H-^{1}H COSY spectrum (CD_3OD) of compound **3**

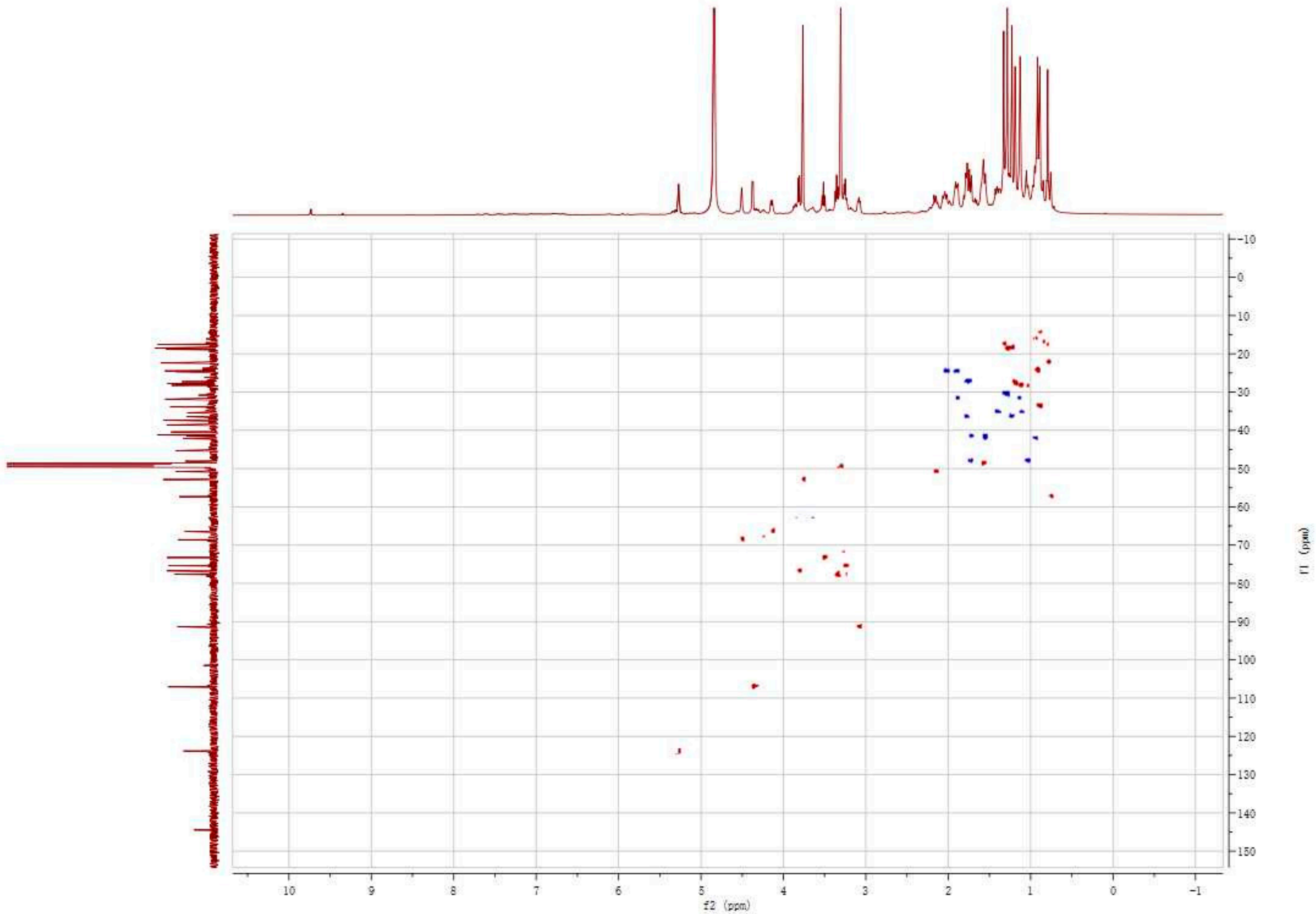

Figure S17. HSQC spectrum (CD_3OD) of compound **3**

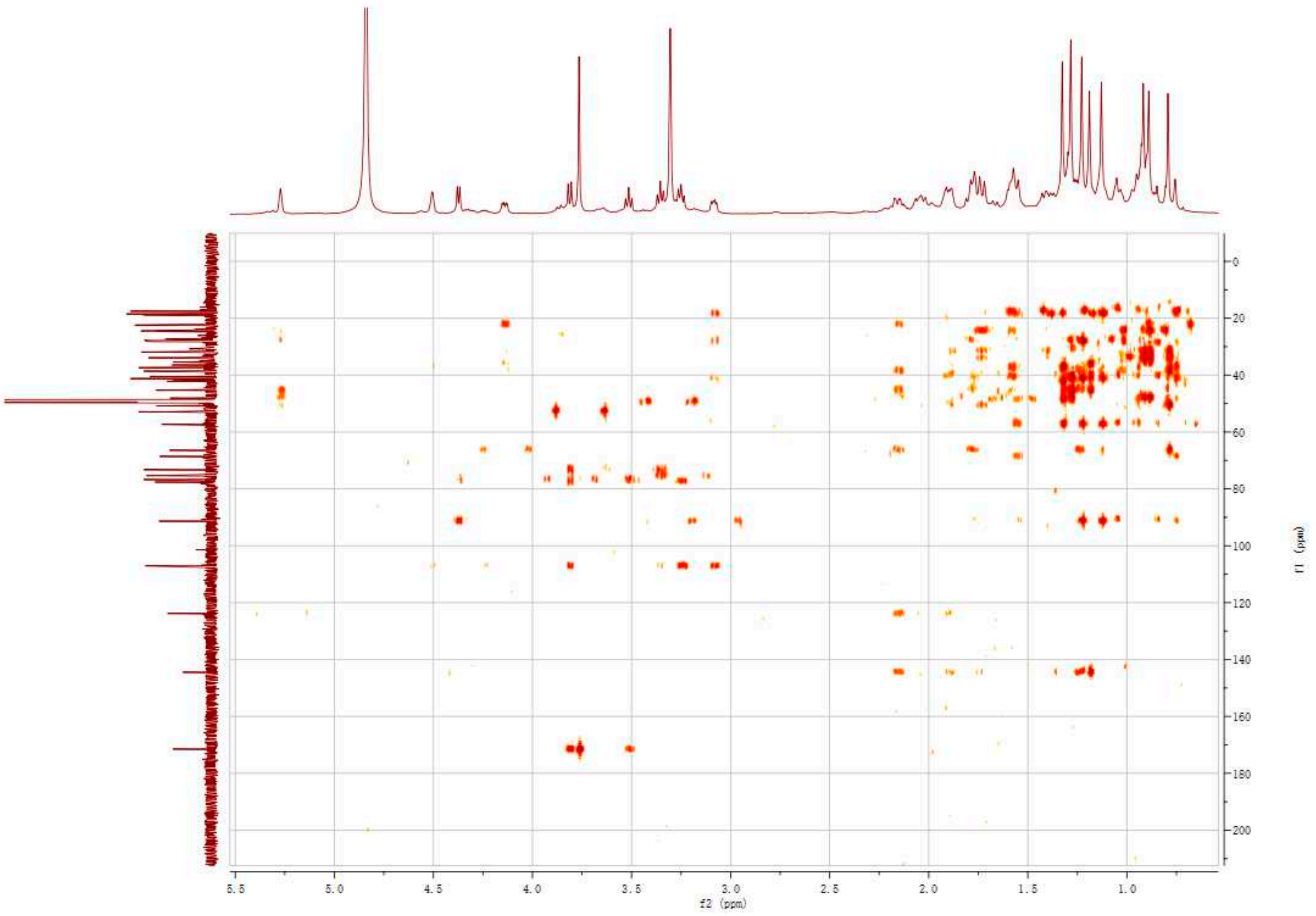

Figure S18. HMBC spectrum (CD_3OD) of compound **3**

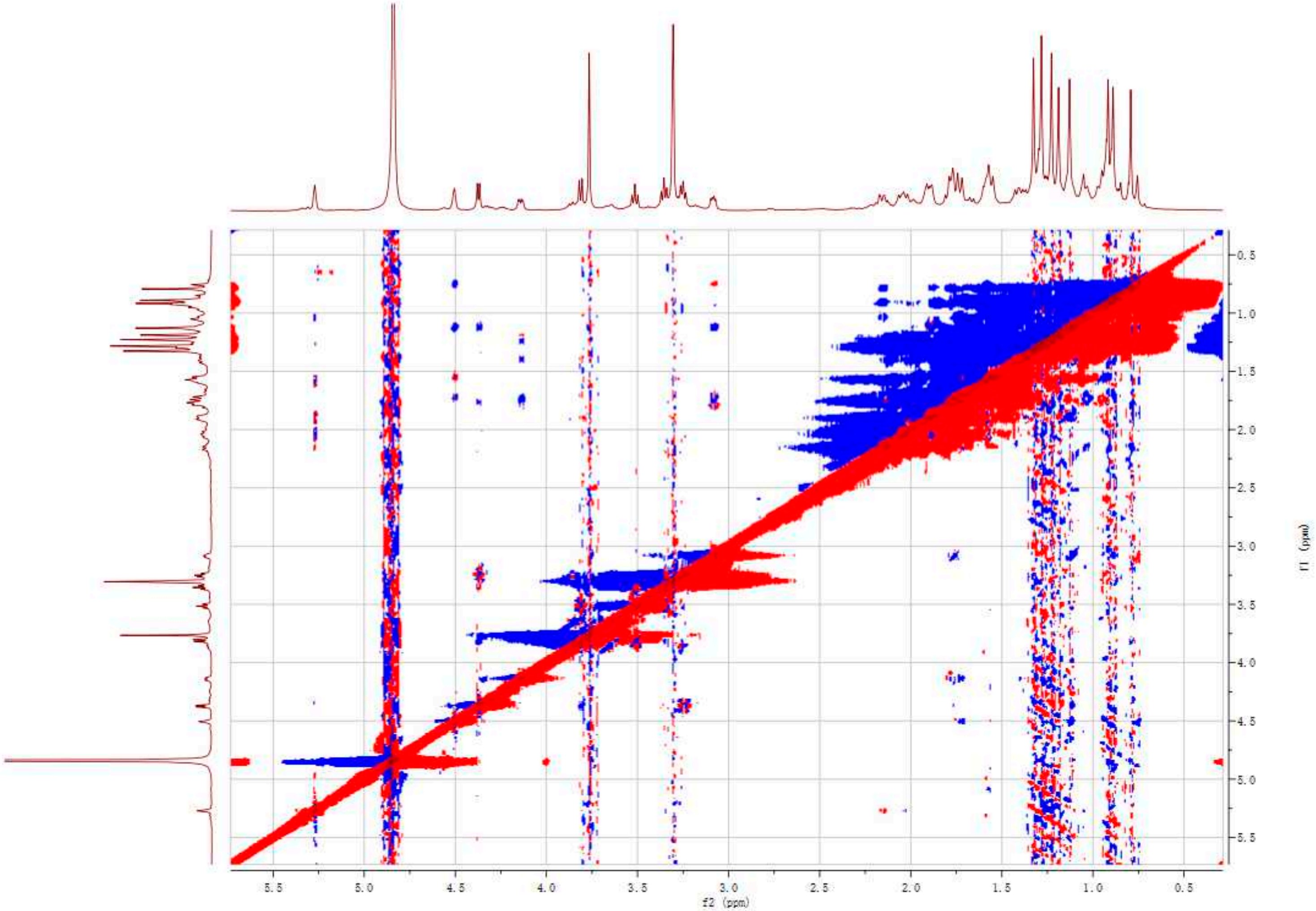

Figure S19. NOESY spectrum (CD_3OD) of compound **3**

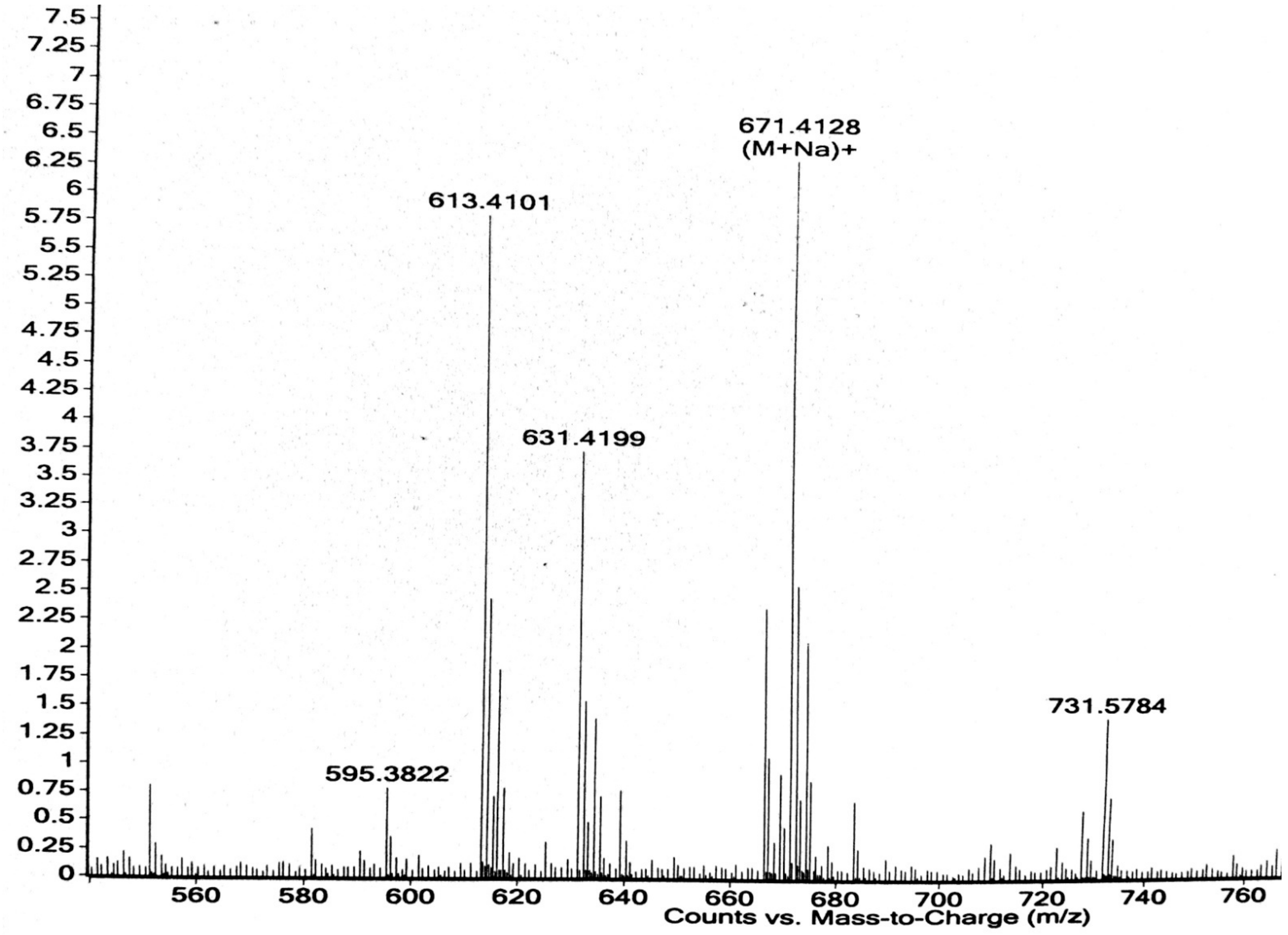

Figure S20. HRESIMS spectrum of compound **3**

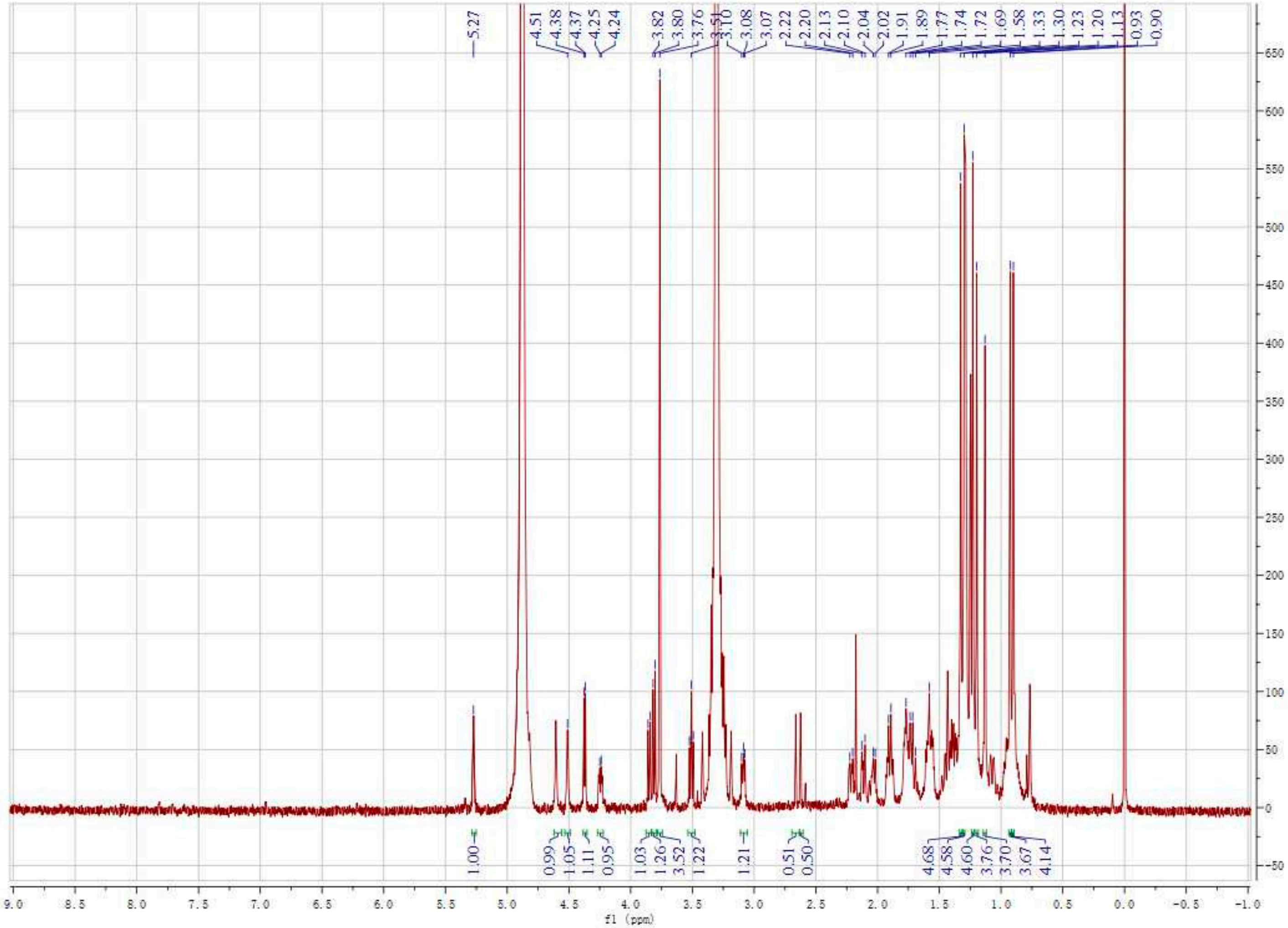

Figure S21. ^{1}H NMR (600 MHz, CD_3OD) spectrum of compound **4**

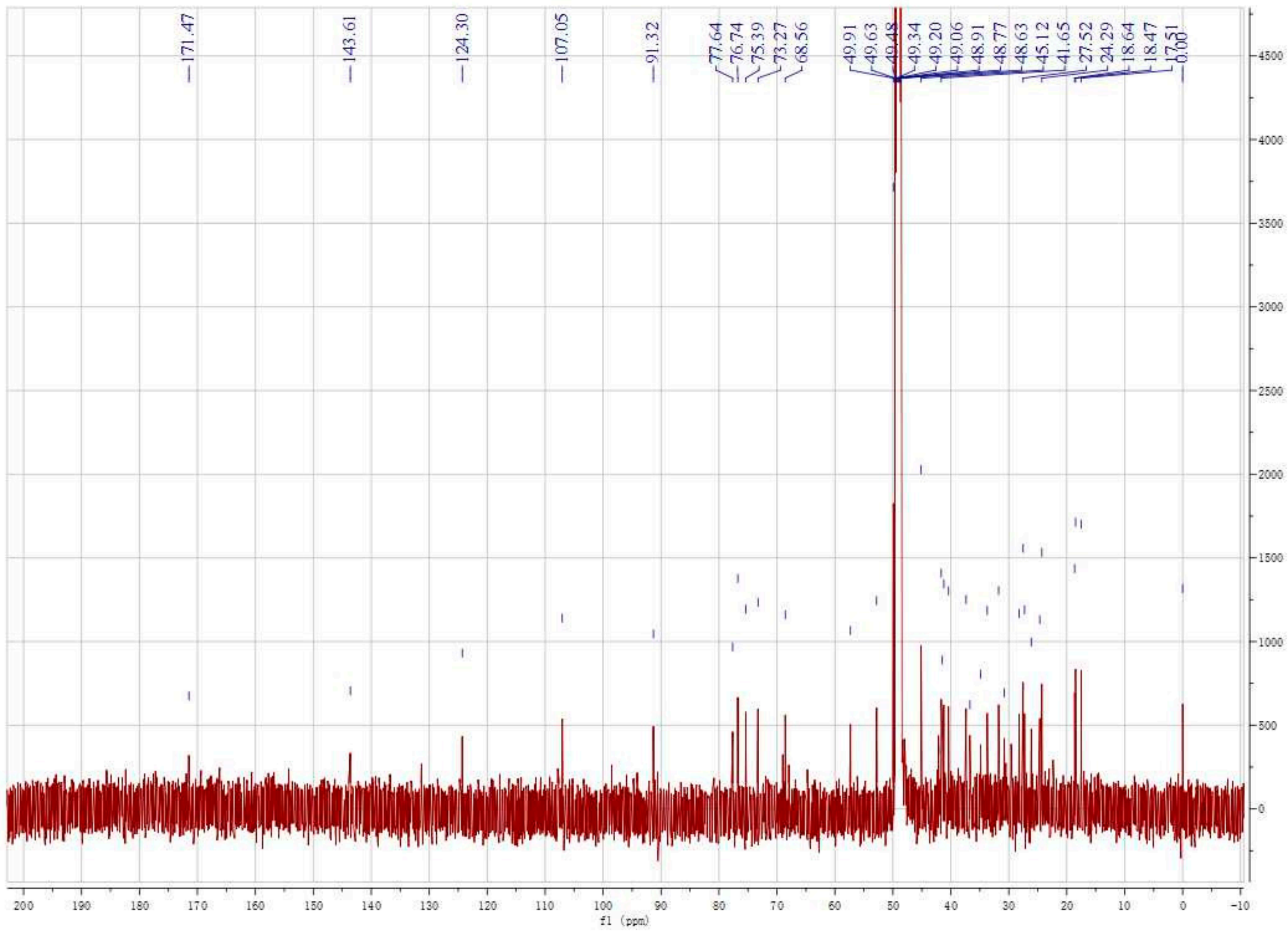

Figure S22. ^{13}C NMR (150 MHz, CD_3OD) spectrum of compound **4**

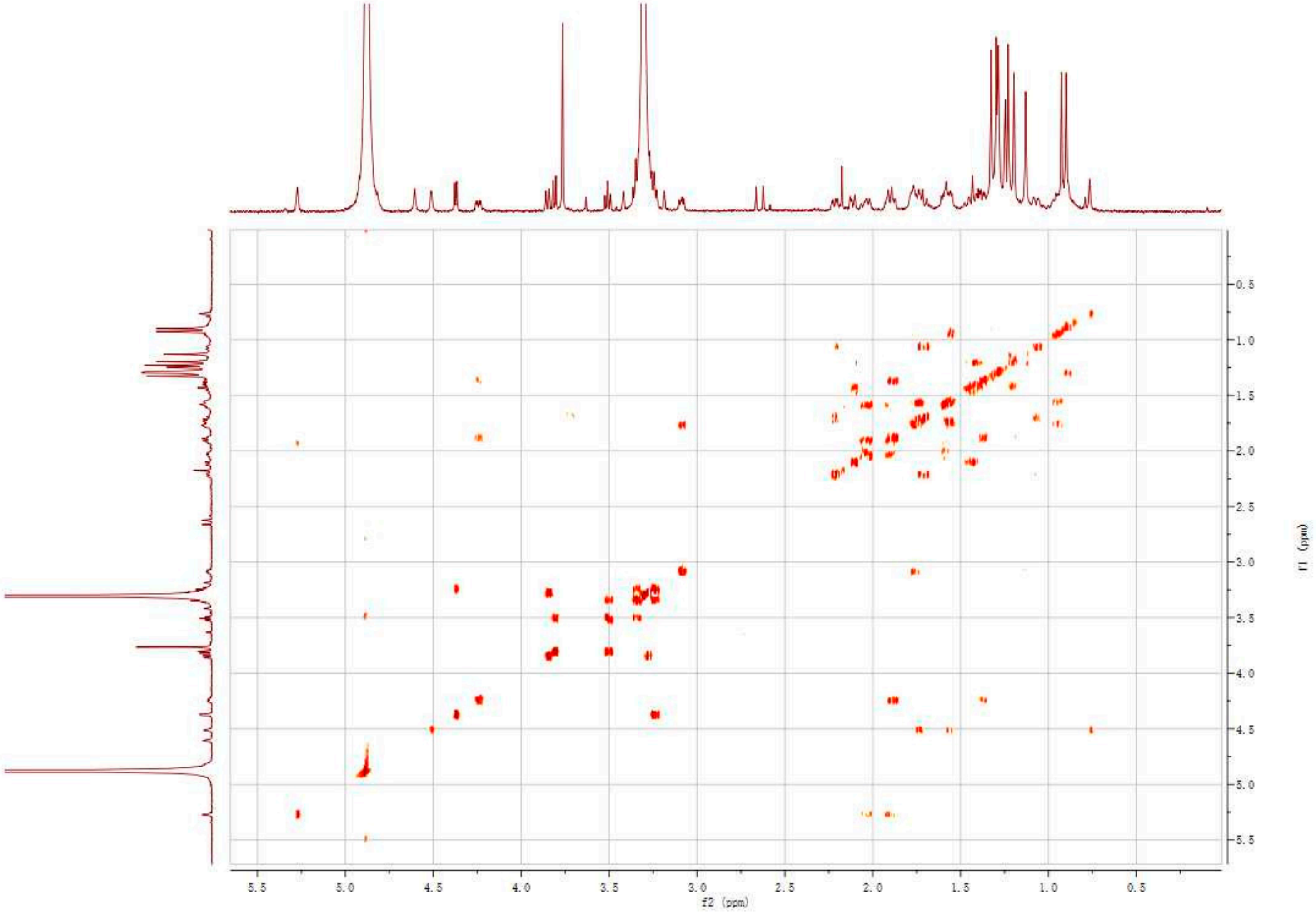

Figure S23. ^{1}H-^{1}H COSY spectrum (CD_3OD) of compound **4**

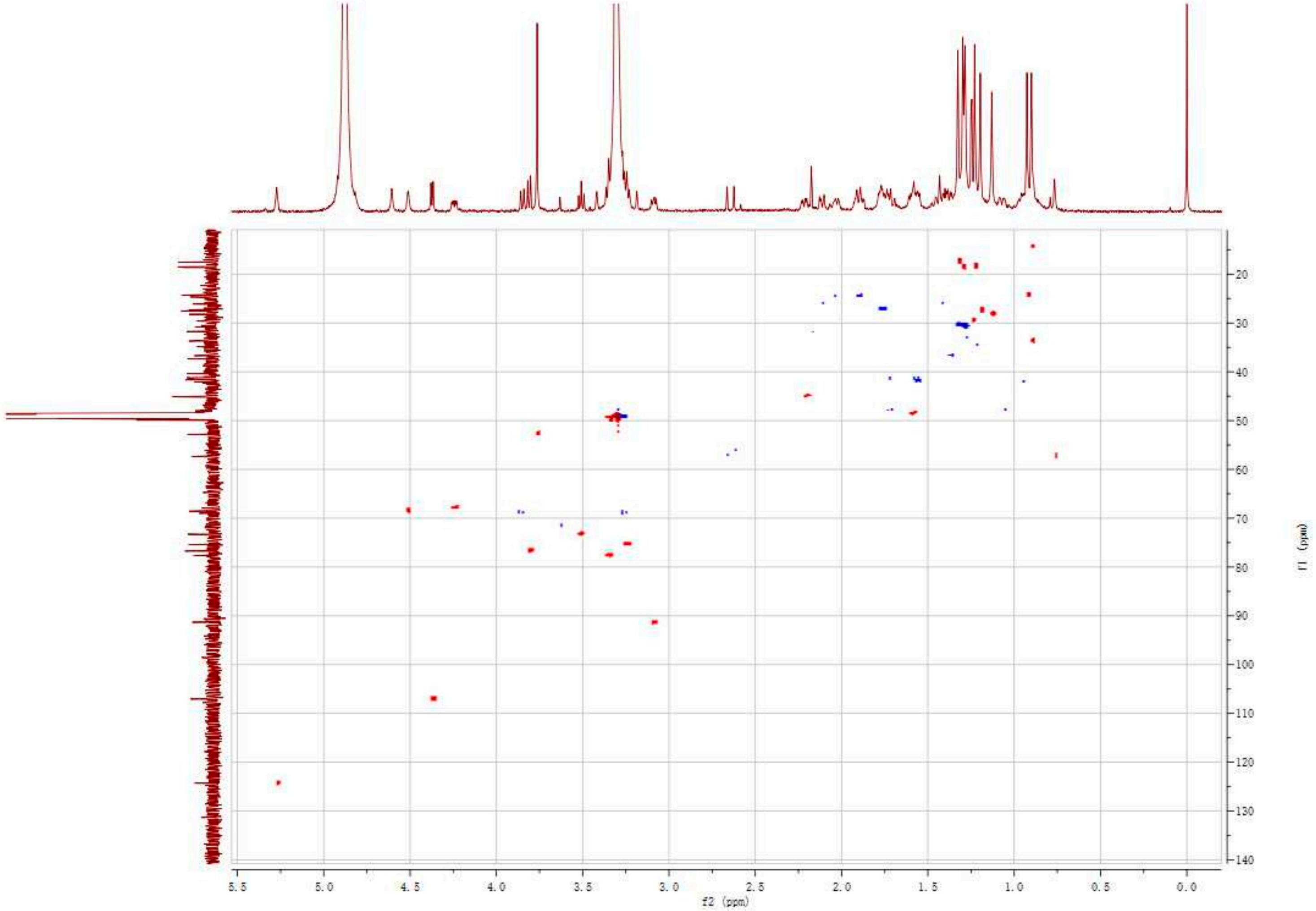

Figure S24. HSQC spectrum (CD_3OD) of compound **4**

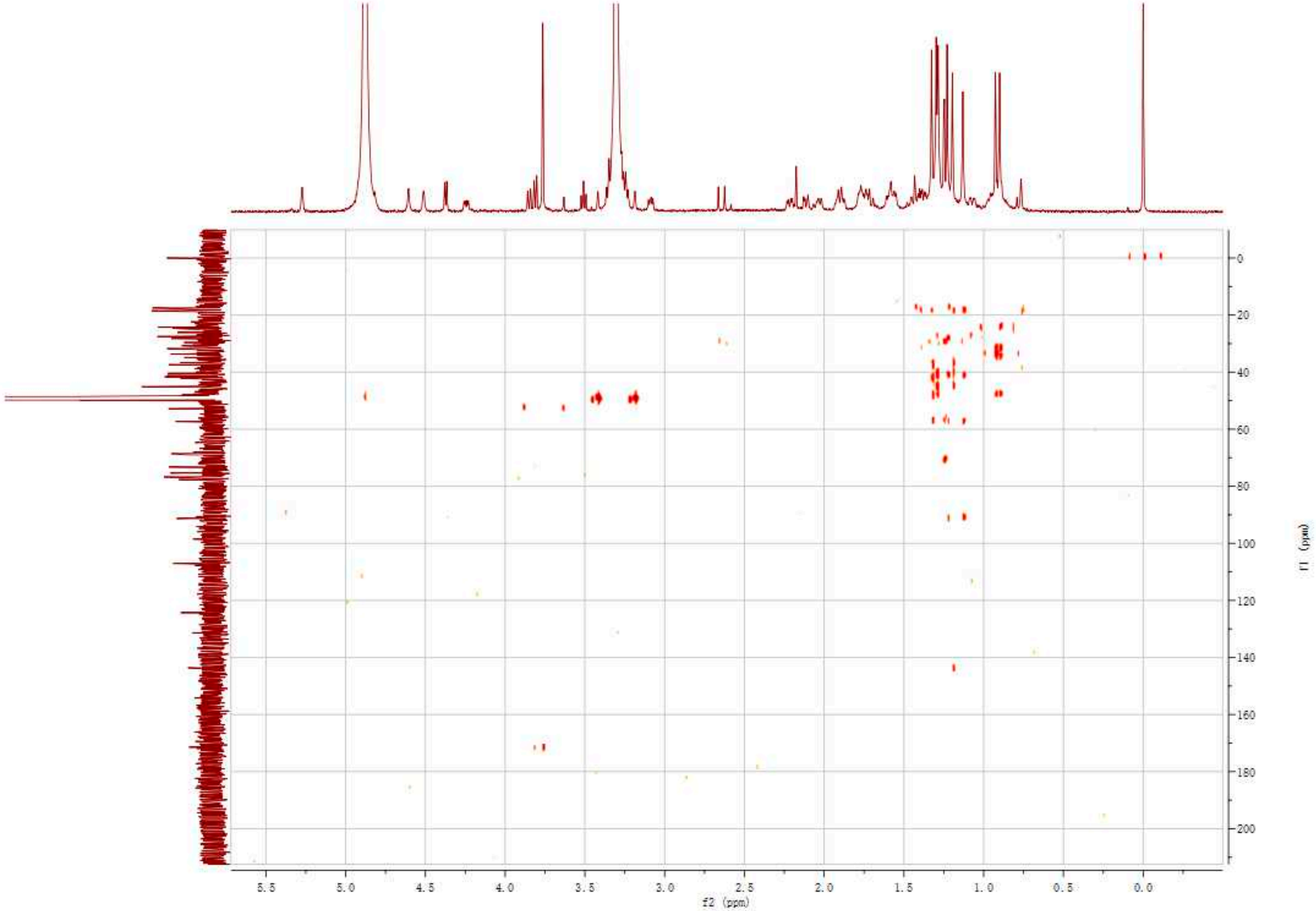

Figure S25. HMBC spectrum (CD_3OD) of compound **4**

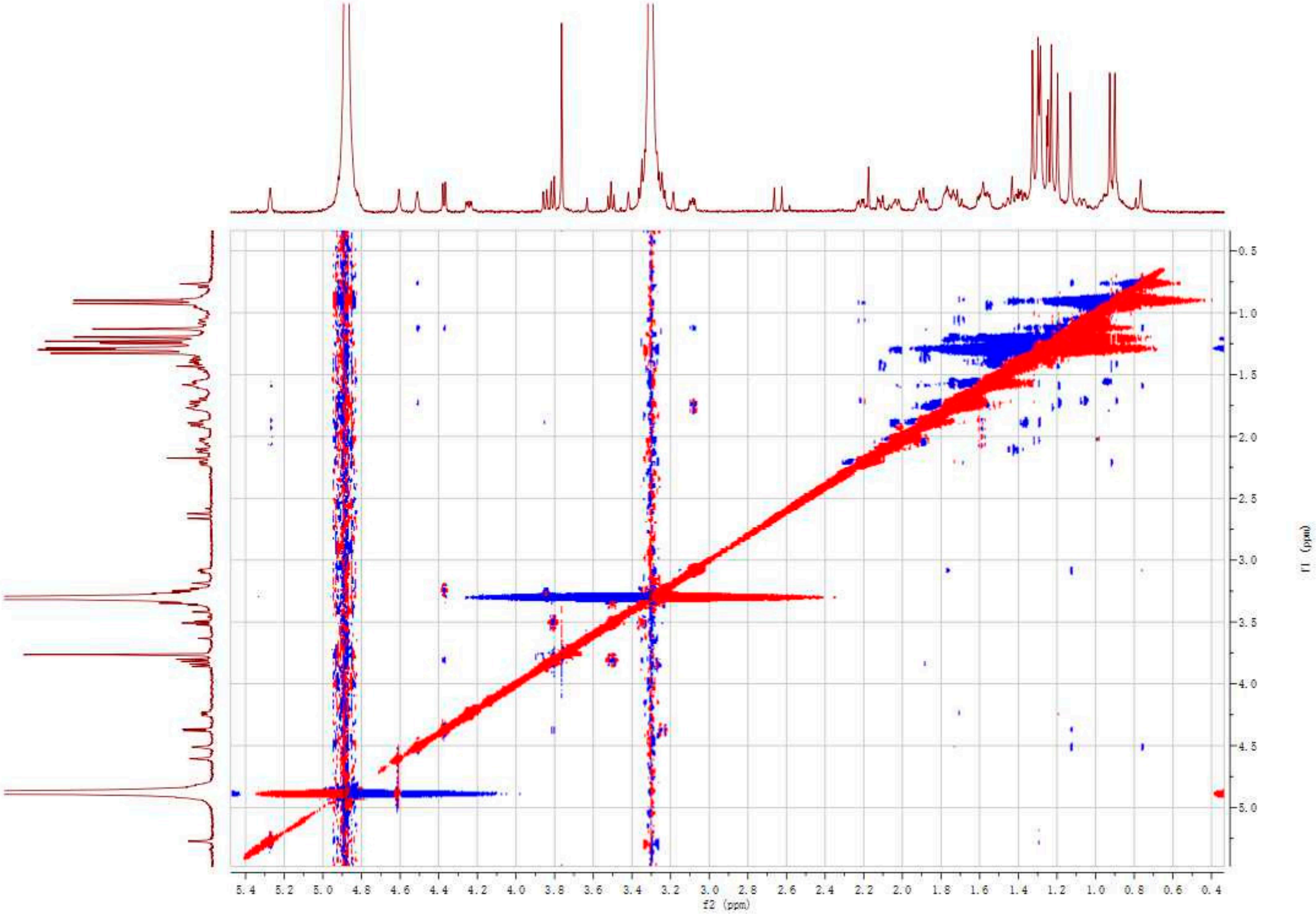

Figure S26. NOESY spectrum (CD_3OD) of compound **4**

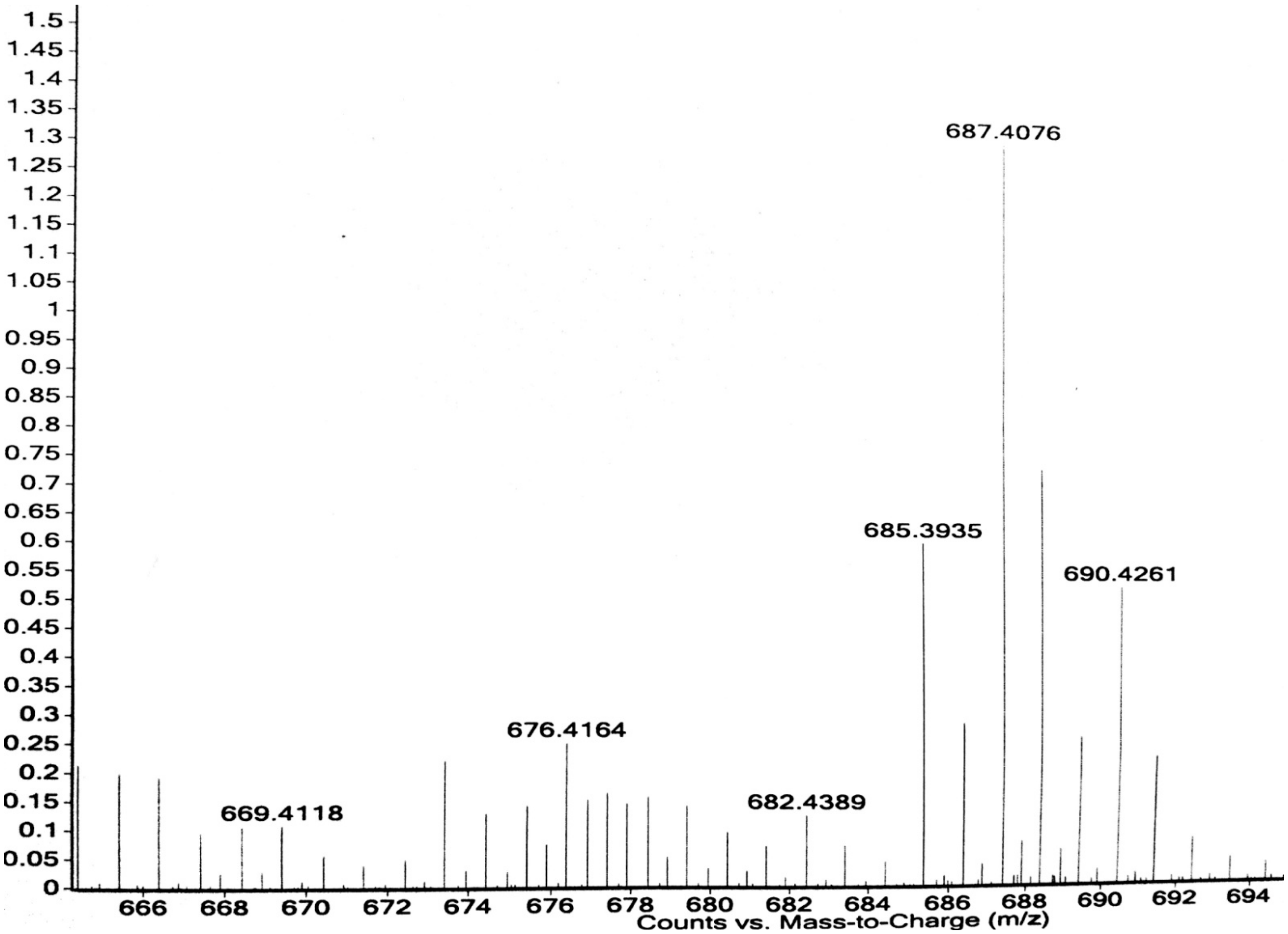

Figure S27. HRESIMS spectrum of compound **4**

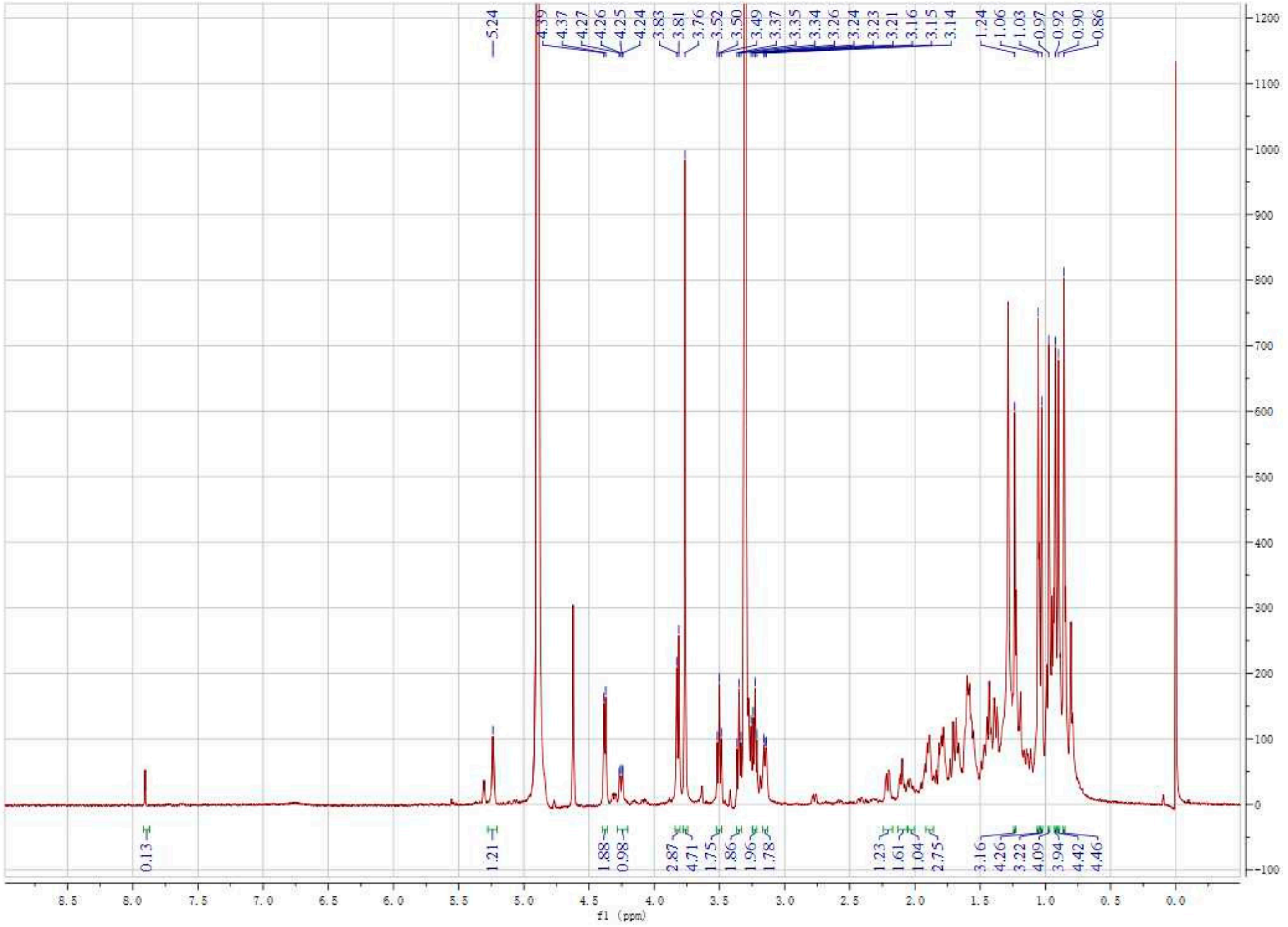

Figure S28. ^{1}H NMR (600 MHz, CD_3OD) spectrum of compound **5**

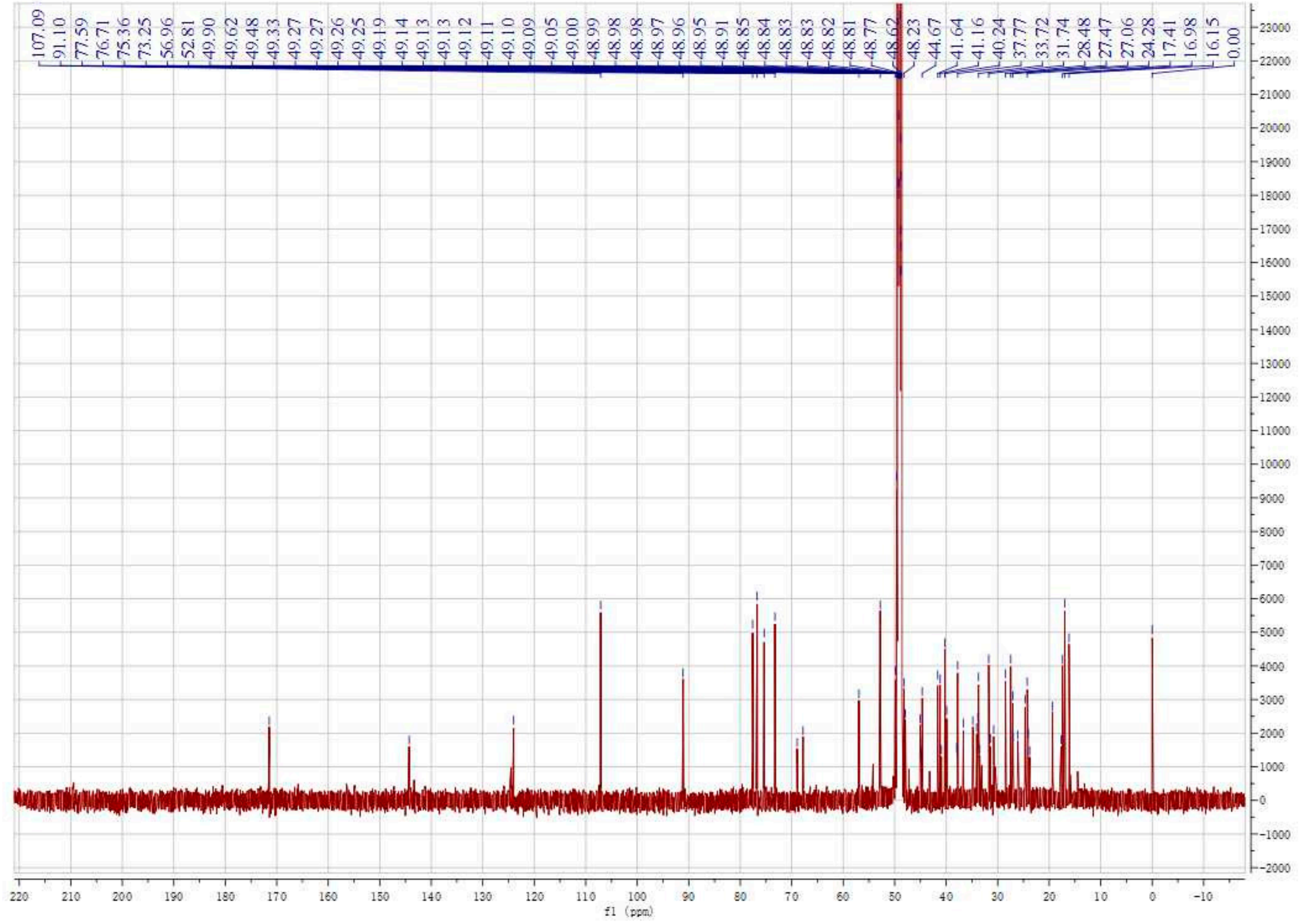

Figure S29. ^{13}C NMR (150 MHz, CD_3OD) spectrum of compound **5**

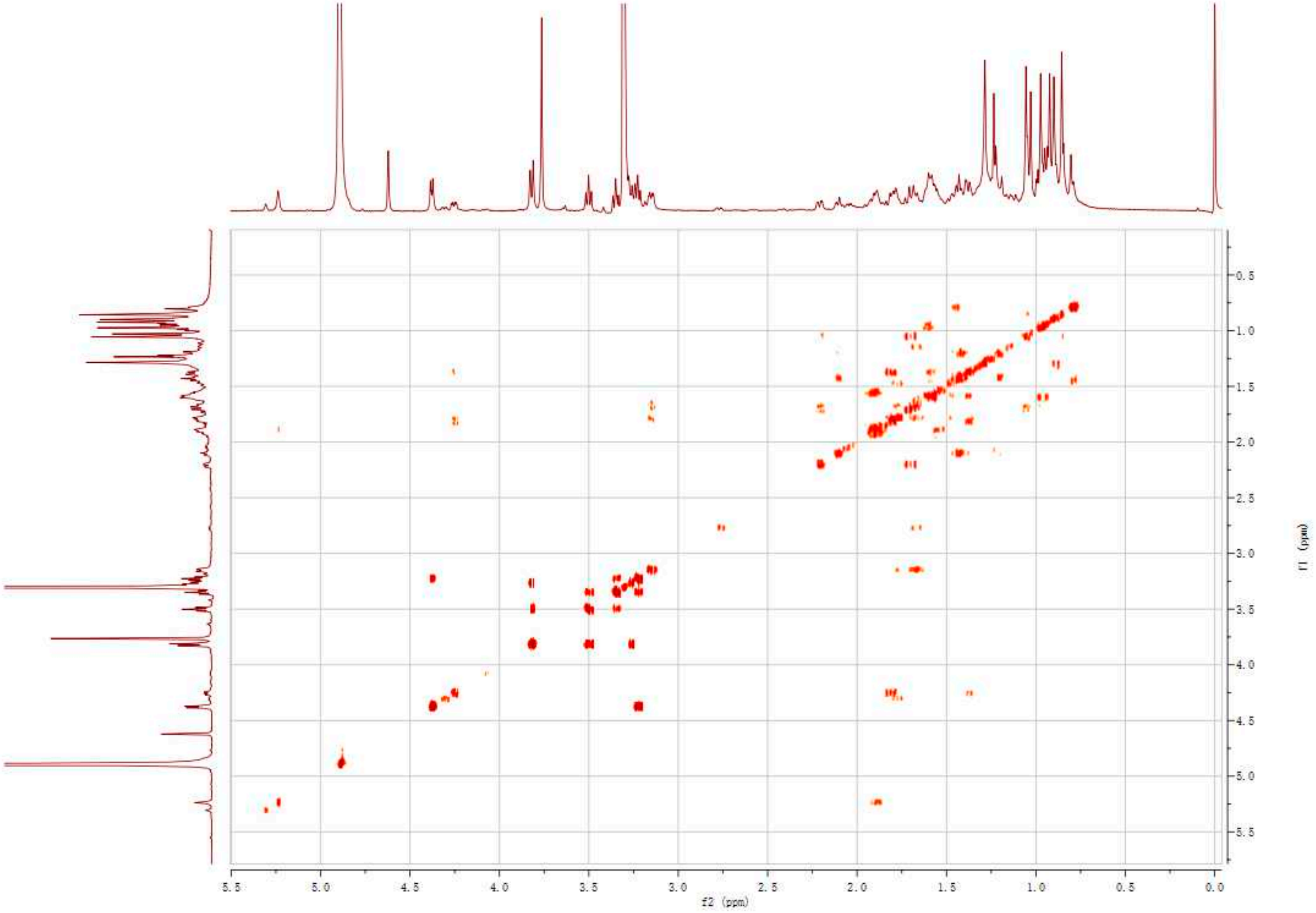

Figure S30. ^{1}H-^{1}H COSY spectrum (CD_3OD) of compound **5**

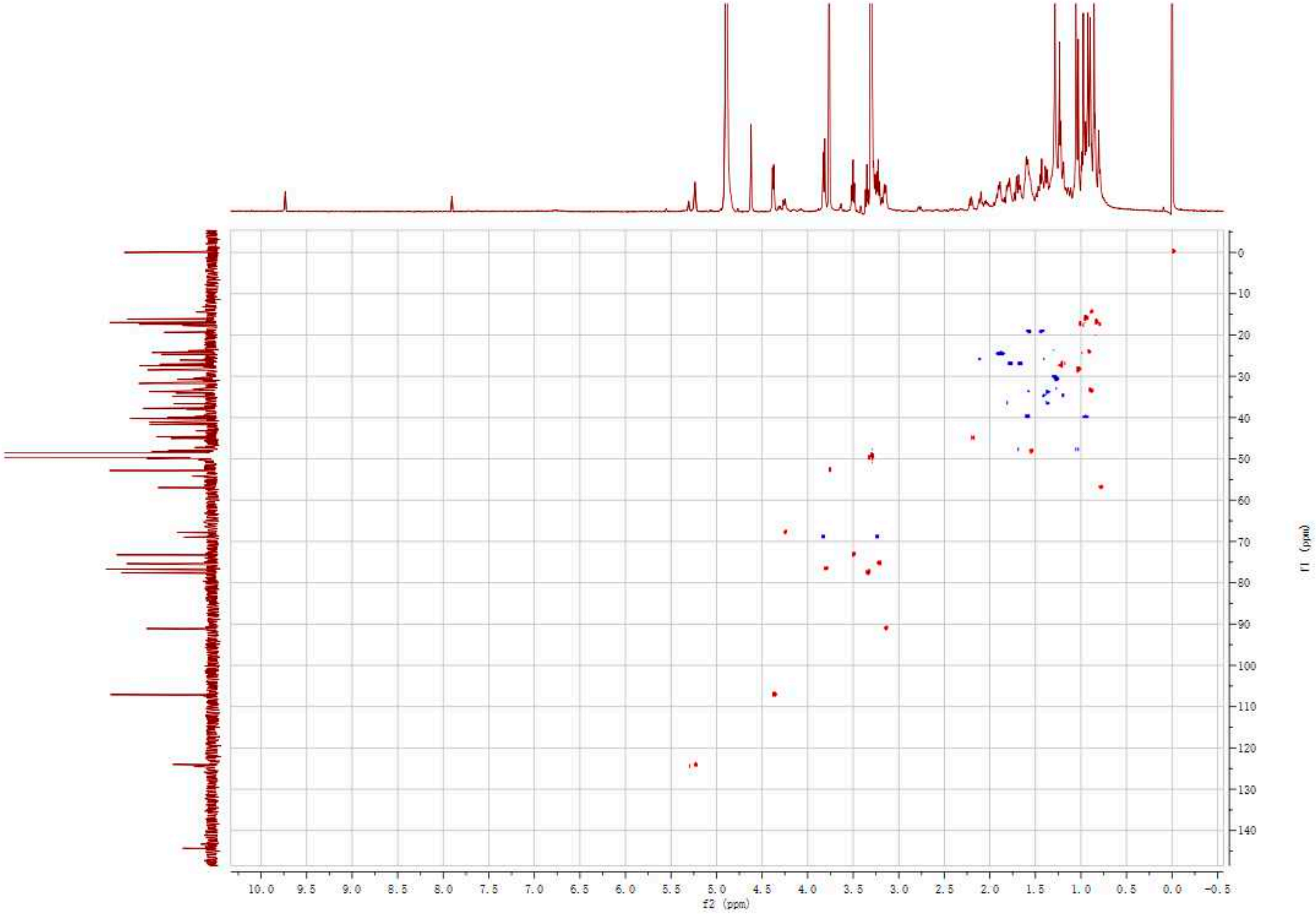

Figure S31. HSQC spectrum (CD_3OD) of compound **5**

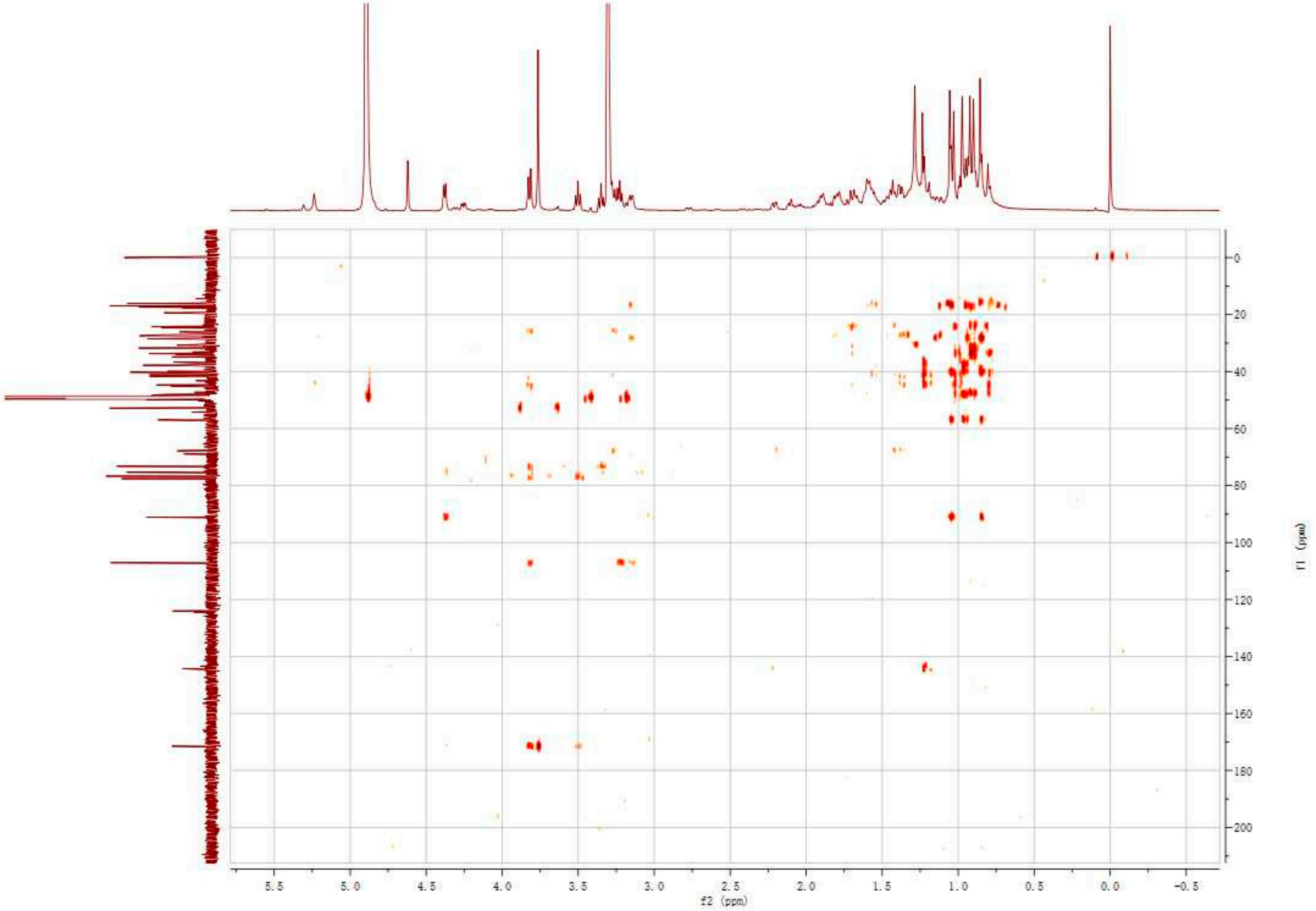

Figure S32. HMBC spectrum (CD_3OD) of compound **5**

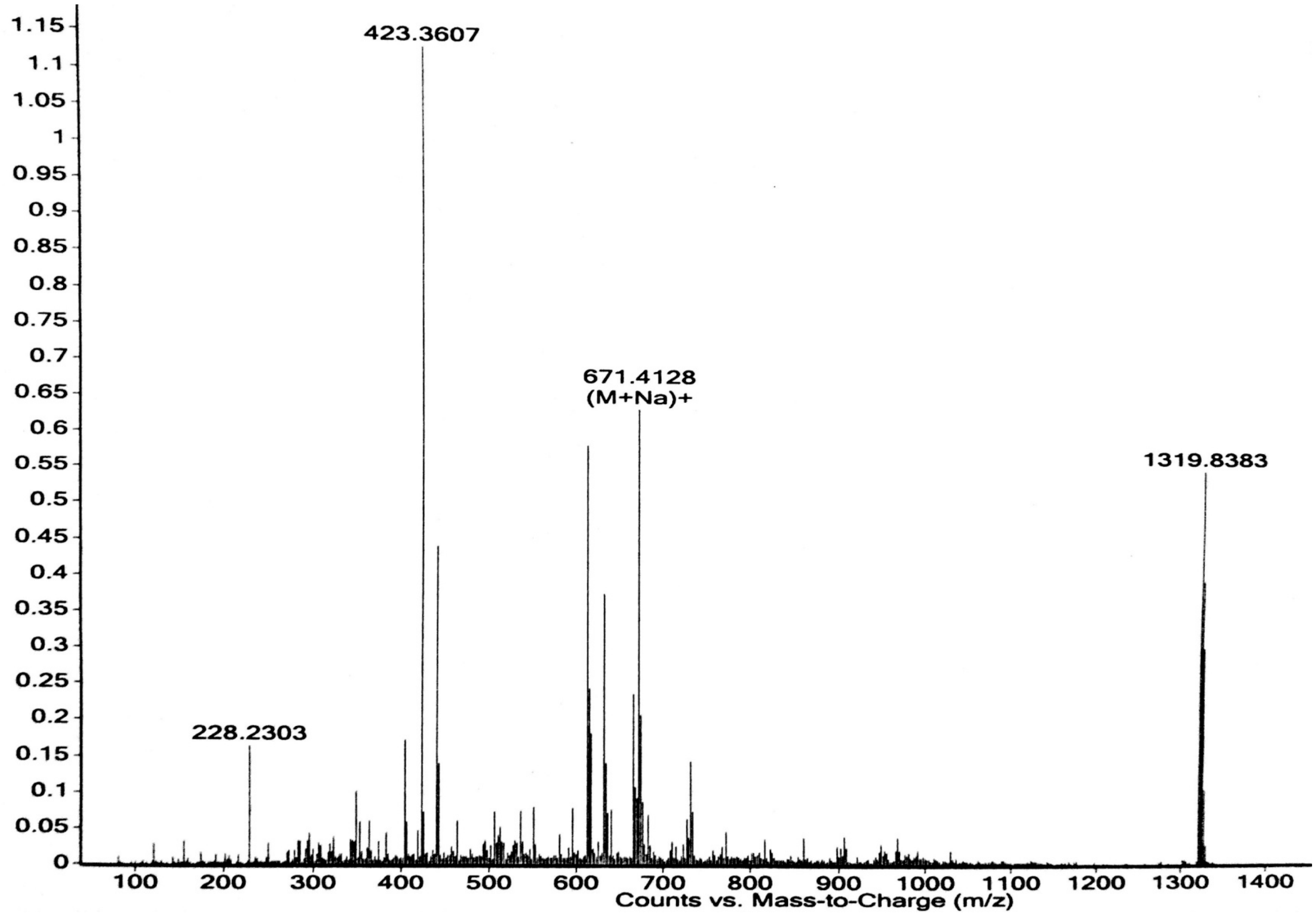

Figure S33. HRESIMS spectrum of compound **5**
